# Permethyl Cobaltocenium (Cp^*^_2_Co^+^) as an Ultra-Stable Cation for Polymer Hydroxide-Exchange Membranes

**DOI:** 10.1038/srep11668

**Published:** 2015-06-29

**Authors:** Shuang Gu, Junhua Wang, Robert B. Kaspar, Qianrong Fang, Bingzi Zhang, E. Bryan Coughlin, Yushan Yan

**Affiliations:** 1 Department of Chemical & Biomolecular Engineering, Center for Catalytic Science and Technology, University of Delaware, Newark, Delaware 19716, USA; 2 Department of Polymer Science and Engineering, University of Massachusetts, Amherst, Massachusetts 01003, USA

## Abstract

Hydroxide (OH^−^)-exchange membranes (HEMs) are important polymer electrolytes enabling the use of affordable and earth-abundant electrocatalysts for electrochemical energy-conversion devices such as HEM fuel cells, HEM electrolyzers, and HEM solar hydrogen generators. Many HEM cations exist, featuring desirable properties, but new cations are still needed to increase chemical stability at elevated temperatures. Here we introduce the permethyl cobaltocenium [(C_5_Me_5_)_2_Co(III)^+^ or Cp^*^_2_Co^+^] as an ultra-stable organic cation for polymer HEMs. Compared with the parent cobaltocenium [(C_5_H_5_)_2_Co(III)^+^ or Cp_2_Co^+^], Cp^*^_2_Co^+^ has substantially higher stability and basicity. With polysulfone as an example, we demonstrated the feasibility of covalently linking Cp^*^_2_Co^+^ cation to polymer backbone and prepared Cp^*^_2_Co^+^-functionalized membranes as well. The new cation may be useful in designing more durable HEM electrochemical devices.

Polymer hydroxide (OH^−^)-exchange membranes (HEMs) are attractive electrolytes for electrochemical energy conversion devices such as fuel cells^1^,^2^,^3^,^4^,^5^,^6^,^7^,^8^,^9^,^10^,^11^,^12^,^13^,^14^,^15^,^16^,^17^,^18^, electrolyzers^19^,^20^, and solar hydrogen generators^21^, largely due to their intrinsic compatibility with non-precious-metal catalysts^2^,^22^ and superior CO_2_ tolerance^23^ compared to liquid alkaline electrolytes^24^. The active hydroxide-conducting component of an HEM is a cation that is covalently linked to a polymer backbone. Organic cations based on nitrogen [ammonium^25^,^26^, pyridinium^27^, guanidinium^28^,^29^, imidazolium^6^,^30^], phosphorus [phosphonium^3^,^8^], sulfur [sulfonium^31^], and ruthenium [bis(terpyridine)ruthenium^7^] have been introduced, featuring specific HEM properties including improved solubility^3^, enhanced thermal stability^31^, and increased basicity^32^. New cations with chemical stability at elevated temperatures (>80 °C^33^,^34^) are still desired to reduce CO_2_ poisoning, increase catalyst activity, and improve heat management in HEM electrochemical devices.

Alkali metal cations (*e.g.*, Li^+^, Na^+^, and K^+^) provide the highest OH^−^ conductivity and show excellent stability, but at present cannot be covalently tethered to a polymer backbone for HEM applications^35^. Organic bis(cyclopentadienyl) metallocenium cations based on VIIIB family metals [(C_5_H_5_)_2_M(III)^+^ or Cp_2_M(III)^+^, M = Co, Rh, Ir, or Mt] satisfy the 18-valence electron stability rule and resemble alkali metal cations: They bear one unit of positive charge, may be precipitated by the addition of excess anions, and with hydroxide as counter-ion absorb CO_2_ and water from ambient air^36^. In particular, cobalt is the smallest atom in the VIIIB family and forms the strongest metal-ring bonds, resulting in the most stable metallocenium cation, Cp_2_Co^+^ (cobaltocenium)^37^. While Cp_2_Co^+^ has been used in strongly basic anion-exchange resins^38^,^39^ and water-soluble redox-active oligomers/polymers^40^,^41^, none of these compounds appears suitable for HEM applications. In addition, the stability of Cp_2_Co^+^ may be still limited due to its unsubstituted Cp rings.

Here we introduce the concept of using permethyl cobaltocenium [(C_5_Me_5_)_2_Co(III)^+^ or Cp^*^_2_Co^+^, Fig. 1a,b] as a highly stable cation for polymer HEMs that are required in designing able and durable electrochemical devices.

## Results

### Cation stability: Cp^*^_2_Co^+^ vs. Cp_2_Co^+^

Complete methylation of the Cp ring in Cp^*^_2_Co^+^ enhances electron donation to the metal center, strengthening the metal-ring bond and delocalizing positive charge away from the metal center^42^. Calculations confirm that Cp^*^_2_Co^+^ has a lower heat of formation (Δ*H*_f_) than Cp_2_Co^+^, suggesting stronger bonding: 499 *vs.* 775 kJ mol^−1^ (a 36% reduction), as predicted by the semi-empirical quantum chemistry software MOPAC2012 (Table 1). The same calculation confirms that the charge on cobalt (*δ*_Co_) is lower in Cp^*^_2_Co^+^ than in Cp_2_Co^+^: +0.988 *vs.* +1.058 *e* (a 6.6% reduction). (Note that the overall system charge remains exactly +1 for both cations, regardless of delocalization.) This reduced charge is consistent with the substantial negative shift (*ca.* 600 mV) in redox potential (*φ*) observed for Cp^*^_2_Co(III)^+^/Cp^*^_2_Co(II) *vs.* Cp_2_Co(III)^+^/Cp_2_Co(II) (−1.24 V *vs.* −0.63 V, referring to the standard hydrogen electrode, SHE, with CH_2_Cl_2_ as solvent^43^).

The reduced positive charge on the metal center makes Cp^*^_2_Co^+^ less susceptible to nucleophilic attack by hydroxide, which is, in general, the first step in HEM degradation. In addition to the electronic effect, steric hindrance^42^ from the methyl groups in the Cp^*^ ligands may also further protect Cp^*^_2_Co^+^ from hydroxide attack. The difference in steric hindrance between Cp^*^_2_Co^+^ and Cp_2_Co^+^ can be quantified^6^ by comparing the accessible angle (*θ*) that is formed by the cobalt center and the edges of circumcircle of hydrogen atoms (Fig. S1). Cp_2_Co^+^ has an accessible angle of 72.4° while Cp^*^_2_Co^+^ has much smaller accessible angles of 40.3–49.6° (Table 1). The smaller accessible angle reduces the chance for the cobalt atom to be attacked.

**Figure 2:**
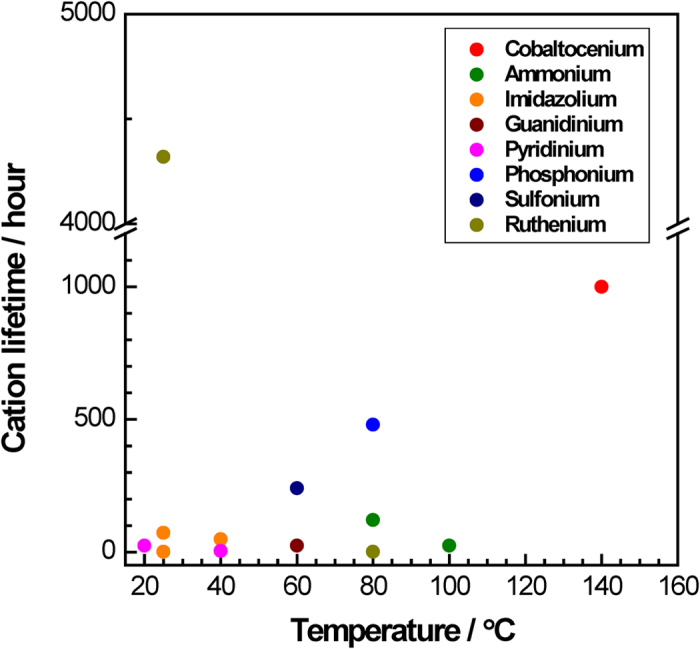
Alkaline stability of Cp^*^_2_Co^+^ and other reported cations. Test conditions: 1 M KOD or NaOD in D_2_O, 20% degradation threshold on ^1^H NMR basis (unless otherwise noted). Cobaltocenium: Cp^*^_2_Co^+^ (^13^C NMR spectroscopy, this work). Ammonium: benzyl-trimethylammonium (*btm*Am) [80 °C in 1 M NaOD/(D_2_O+CD_3_OD)^8^ and 100 °C in 1 M NaOD/D_2_O, this work]. Imidazolium: benzyl-1-methyl-limidazolium (*bm*Im)^67^, 1,3-dimethyl-2-phenyl-benzimidazolium (*dmp*BIm) (0.3 M KOH)^6^, 1,3-dimethyl-2-(2,4,6-trimethylphenyl)-benzimidazolium (*dmtmp*BIm) (1.3 M KOH)^6^. Guanidinium: benzyl-pentamethylguanidium (*bpm*Gu) (this work). Pyridinium: benzylpyridinium (*b*Py) (this work). Phosphonium: tetrakis(dialkylamino)phosphonium (*tkdaa*Ph) [1 M NaOD/(D_2_O+CD_3_OD)]^8^. Sulfonium: (4-methoxyphenyl)-diphenylsulfonium (*mopdp*Su)^31^. Ruthenium: bis(terpyridinine)ruthenium (*ttp*Ru) (UV-vis spectroscopy). The chemical structures of those cations are shown in Table S3. ^1^H NMR spectra of *btm*Am, *bpm*Gu, and *b*Py are not shown.

Indeed, a high-temperature alkaline stability test (at 140 °C in 1 M NaOD/D_2_O) showed that Cp^*^_2_Co^+^ is significantly more stable than Cp_2_Co^+^. After six weeks (1,000 hours) only 8.5% of the initial Cp^*^_2_Co^+^ had been found to degrade (^13^C NMR, Fig. S2) whereas Cp_2_Co^+^ degraded completely after only one week (Fig. S3). Further, Cp^*^_2_Co^+^ showed no change in UV-vis absorption after the stability test (Fig. S4). For comparison, a typical ammonium cation (trimethyl benzylammonium) degraded by 18% in 24 hours (Fig. S5). Cp^*^_2_Co^+^ demonstrates a level of stability that has not been achieved by any known HEM cations (Fig. 2).

The reduced charge on cobalt also improves the basicity of the cation hydroxide, since weakened cation-anion interaction favors dissociation. Indeed, the base dissociation constant (*K*_b_) is much larger for Cp^*^_2_Co^+^OH^−^ than for Cp_2_Co^+^OH^−^: p*K*_b_ = 2.7 *vs.* 5.4 (a 500-fold increase in *K*_b_, Table 1), as measured by correlating OH^−^ concentration with total organic base concentration in aqueous solution (Table S1 and Fig. S6). Improved basicity is expected to increase the hydroxide conductivity of the corresponding HEMs.

Superior stability and basicity suggest that Cp^*^_2_Co^+^ is a much more desirable cation than Cp_2_Co^+^ for HEM applications.

### Synthetic strategy for Cp^*^_2_Co^+^-PSf polymer

Cp^*^_2_Co^+^-PSf was synthesized by a diamine-bridge strategy (Fig. S7). The feasibility of this approach was established by a series of small-molecule model reactions (bromination, Fig. S8; anion exchange, Fig. S9; amination, Fig. S10; and cation incorporation, Fig. S11), in which benzyl chloride functioned as a surrogate for the halomethylated polymer. The Cp^*^_2_Co^+^ cation was first brominated to introduce a single halomethyl group. In addition to ^1^H NMR spectroscopy (Fig. S8), the bromination step was further confirmed by ^13^C NMR (Fig. S12) and mass spectroscopy (Fig. S13, no sign of multiple bromination). In parallel, one amine group from hexamethylenediamine (HMDA) was linked to benzyl chloride by amination (leaving the other amine group intact). Then, the remaining unreacted amine group from HMDA was used to link the brominated Cp^*^_2_Co^+^. Reaction conditions for all steps were optimized (yield >95%, ^1^H NMR spectroscopy).

In a similar manner, Cp^*^_2_Co^+^-PSf was synthesized (Figs. S14 and S15) and then membranes were prepared. Unlike the small-molecule model reaction, proper reaction conditions are critical for amination of polysulfone. Firstly, a low reaction temperature (*e.g.*, 20 °C) avoids the ammonolysis that leads to polysulfone depolymerization (observed at 40 °C and higher). Secondly, a large excess of amine (*e.g.*, 20 equiv.) avoids cross-linking in which one amine molecule reacts with two chloromethyl groups. Once cross-linked, the polymer becomes insoluble, and is no longer suitable for further reactions.

**Figure 3:**
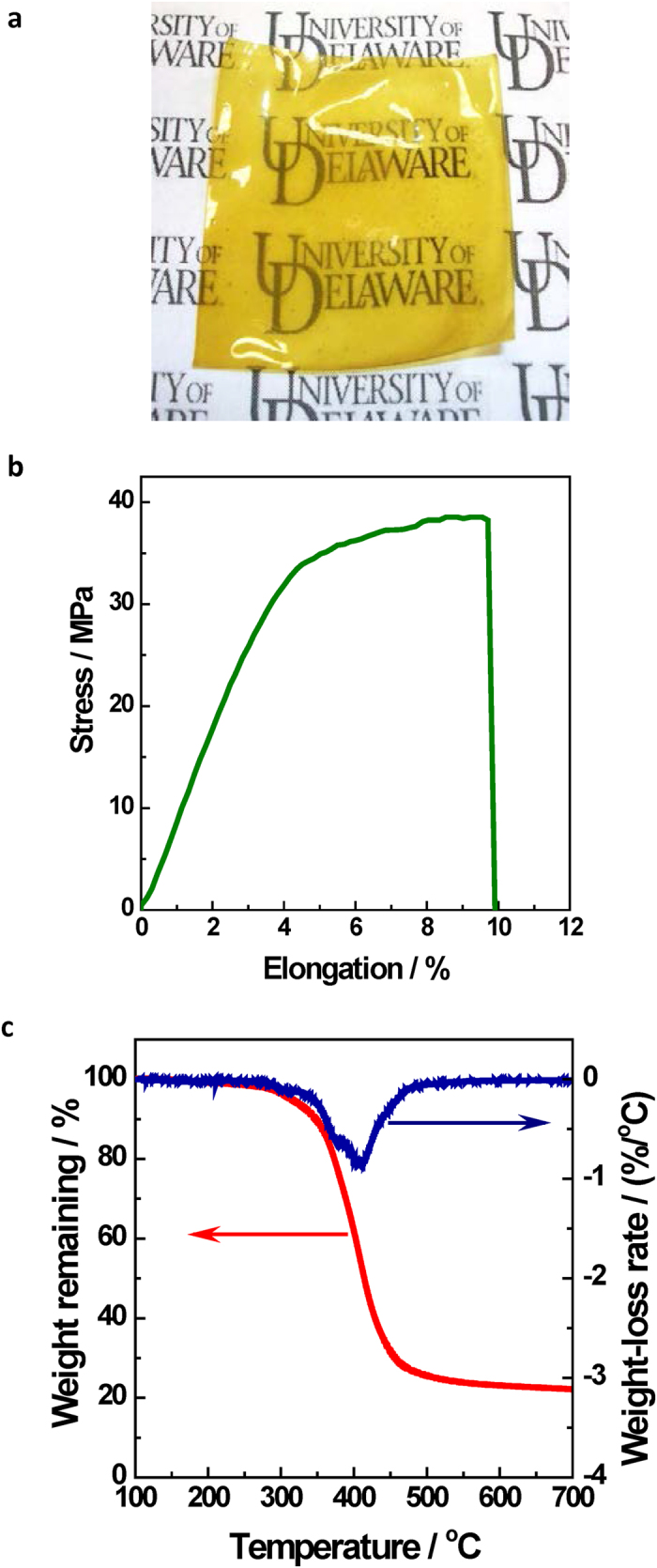
Characterization of Cp^*^_2_Co^+^-PSf membrane. (**a**) Photograph (2” x 2”, 100 μm thick). (**b**) Dynamic mechanical analysis (DMA) test curve (ambient humidity and temperature, 10 mm min^−1^ cross-head speed). (**c**) Thermal gravimetric analysis (TGA) and derivative thermal gravimetric (DTG) curves (10 °C min^−1^, nitrogen atmosphere).

Note that the hydrophobic^44^,^45^ PF_6_^−^ anion in Br-Cp^*^_2_Co^+^PF_6_^−^ must be exchanged for a hydrophilic anion (*e.g.*, Cl^−^) prior to linking the cation to the backbone, due to the difficulty of exchanging PF_6_^−^ for the ultimately desired OH^−^ in polyelectrolytes^7^. The counter-ion was later exchanged from Cl^−^ to OH^−^ with 1 M KOH. The Cp^*^_2_Co^+^-PSf membranes were found be to flexible, uniform in thickness, and transparent (Fig. 3a).

### Microstructure and properties of Cp^*^_2_Co^+^-PSf membranes

Due to the presence of cobalt, high-contrast transmission electron microscopy (TEM) images were obtained without the need to employ a dye anion (*e.g.*, PdCl_4_^2−^ 46 or WO_4_^2−^ 16), directly revealing detailed features of the membrane microstructure (Fig. S16a). Overall, the hydrophilic (dark) and hydrophobic (light) domains are homogenously interspersed, which is essential to simultaneously provide ionic conduction and mechanical robustness^16^. The hydrophilic domains show an average size of 15 nm, similar to those in typical ammonium-based membranes (10–30 nm) with the same polysulfone backbone and similar ion exchange capacity (IEC, 1.14 mmol/g)^46^. The presence of cobalt was also confirmed by energy dispersive X-ray (EDX) spectroscopy during imaging (Fig. S16b). High tensile strength (40 MPa with 10% elongation at break, Fig. 3b) shows that the membrane is strong and robust. Under similar test conditions, the commercial proton exchange membrane (PEM) Nafion^®^ 212 is half as strong (around 20 MPa^47^).

Two membranes of different degrees of functionalization (DFs, 100% and 123%) were characterized (Table 2). Both hydroxide conductivity and water uptake increase with ion exchange capacity (IEC), as with HEMs based on other cations. Note that the measured IECs are very close to the theoretical ones: 1.04 *vs.* 1.09 mmol g^−1^ for Cp^*^_2_Co^+^-PSf100 membrane; 1.16 *vs*. 1.20 mmol g^−1^ for Cp^*^_2_Co^+^-PSf123 membrane. Such an agreement indicates the Cp^*^_2_Co^+^ cations in Cp^*^_2_Co^+^-PSf membranes are fully functional. For comparison, HEMs with similar theoretical IEC and similar backbone (when available) but different cations are summarized in Table 2. The Cp^*^_2_Co^+^-PSf membranes are similar to other membranes in terms of hydroxide conductivity (10–22 *vs.* 0.19–45 mS cm^−1^) and water uptake (41%–68% *vs.* 8.2%–240%) in deionized water at room temperature, as well as IEC-normalized hydroxide conductivity (HC_IEC_, 9.2–18 *vs.* 0.15–38 mS∙g cm^−1^ mmol^−1^), suggesting similar cation basicity and hydroxide conduction efficiency. As expected, hydroxide conductivity of Cp^*^_2_Co^+^-PSf membranes increases with temperature. E.g., the hydroxide conductivity of Cp^*^_2_Co^+^-PSf123 membranes reached 49 and 64 mS cm^−1^ at 60 and 80 °C, respectively.

### Thermal and alkaline stability of Cp^*^_2_Co^+^-PSf membranes

The membranes showed very high thermal stability, consistent with the aforementioned stability of the Cp^*^_2_Co^+^ cation. The onset of decomposition temperature of 305 °C (Fig. 3c) is the highest among all reported HEMs: *e.g.*, 60 °C higher than a sulfonium-functionalized PSf^31^, 120 °C higher than a phosphonium-functionalized PSf^48^, or 150 °C higher than ammonium-functionalized PSfs^46^,^49^. Note that the thermal gravimetric analysis (TGA) data only reflect short-term thermal stability and are only useful for comparison under similar test conditions.

The Cp^*^_2_Co^+^-PSf membranes also showed improved alkaline stability: At 80 °C in 1 M KOH, the IEC loss of Cp^*^_2_Co^+^-PSf membranes was 18% and 27% of the initial value after 1,000 and 2,000 hours, respectively (Fig. S17). After the 1,000-hour stability test, solid-state ^13^C NMR spectroscopy confirmed that Cp^*^_2_Co^+^ functional groups remained almost unchanged (Fig. S18), but there were clear signs of PSf backbone scission. This result is consistent with that of a recent study^50^, and it further highlights the need for more stable backbones in developing next-generation highly durable HEMs.

Under a more aggressive test at 100 °C for 2,000 hours, the IEC lost was about 50% for Cp^*^_2_Co^+^-PSf membranes. Considering that higher test temperature leads to shorter membrane lifetime, the membrane lifetime is plotted against the test temperature (Fig. S19). With 20% loss of initial IEC as the failure criterion, Cp^*^_2_Co^+^-PSf membranes are more stable than all other cation-based HEMs reported to date.

## Discussion

Polysulfone was used as the polymer backbone in this work primarily to demonstrate linking Cp^*^_2_Co^+^ to polymer and to compare Cp^*^_2_Co^+^ to other cations reported. However, polysulfone backbone is sensitive for HEM degradation^50^,^51^, and more stable polymer backbones are needed to better match the stable Cp^*^_2_Co^+^ cation. Very recently, alternative polymer backbones, such as polystyrene^52^ and poly(phenylene)^53^, have been shown to have better stability than polysulfone in alkaline media. In principle, the synthesis reported here may be modified to employ those more stable polymer backbones. The incorporation of Cp^*^_2_Co^+^ cation to more stable polymer backbones and the synthesis and stability of their resulting membranes warrant important future research.

In the amination step, the chain length of the diamine linker between the polymer backbone and the Cp^*^_2_Co^+^ cation was found to affect membrane flexibility, likely resulting from the incompatibility between the rigid hydrophobic polymer backbone and the bulky hydrophilic cation. Increasing the chain length (*e.g.*, ethylene^54^, propylene^54^,^55^, and hexamethylene^56^,^57^,^58^) between cation and backbone helps alleviate this incompatibility. Indeed, HMDA, with six carbon atoms and two nitrogen atoms, was found to be a good choice for preparing flexible, robust membranes.

In the cation incorporation step, although Br-Cp^*^_2_Co^+^PF_6_^−^ could react at two possible amine sites — either close to the polymer backbone (the basal secondary amine) and or at the end of side chain (the terminal primary amine) (Fig. S7) — a reaction was only observed at the terminal site, even for the less-hindered model molecule (Fig. S10). Such good selectivity results from strong steric hindrance in the bulky Br-Cp^*^_2_Co^+^PF_6_^−^ molecule and higher nucleophilicity of the terminal amine group (p*K*_a_ of amines’ conjugate acids: 10.21 and 10.21 for the basal amine *vs.* 9.26 and 9.93 for the terminal amine, of HMDA-aminated chloromethylated polysulfone polymer and HMDA-aminated benzyl chloride small molecule, respectively; calculated by the MarvinSketch software, Table S2).

During the model cation durability test, ^13^C instead of ^1^H NMR spectroscopy was used to monitor stability because both Cp^*^_2_Co^+^ and Cp_2_Co^+^ undergo rapid H-D isotopic exchange upon exposure to alkaline media, rendering ^1^H spectroscopy inappropriate for accessing stability^52^. Figs. S20 and S21 show the degree of H-D isotopic exchange is 78% and 29% for Cp^*^_2_Co^+^ and Cp_2_Co^+^, respectively, under the same test conditions (60 °C, 40% KOH/D_2_O in methanol, 30 min). This difference in degree of exchange is consistent with the observation from the literature that methyl protons are slightly more acidic than ring protons in cobaltocenium [p*K*a in methanol: 22.7 for methyl protons of 1,1’-dimethyl-cobaltocenium, (1-Me-Cp)_2_Co^+^, *vs.* 23.9 for ring protons of Cp_2_Co^+^]^59^,^60^.

Table 2 summarizes the HEM samples with similar backbone and IECs for the purpose of comparing different cations. It is noted that HEMs with very high hydroxide conductivity have been reported. In general, high conductivity can be achieved by providing either high IEC or high efficiency of hydroxide conduction. With high IECs (> 2.0 mmol g^−1^), HEMs exhibited high hydroxide conductivities (at ~20 °C unless otherwise noted) such as 43, 50, 65, 67, and 68.7 mS cm^−1^ for a *badm*Am-based PPO (2.75 mmol g^−1^)^9^, a *btm*Am-based fluorenyl-containing poly(ether sulfone ketone) (2.54 mmol g^−1^, 30 °C)^61^, a *btm*Am-based di-fluorinated bpPSf (2.62 mmol g^−1^)^62^, a *bpm*Gu-based bpPSf (2.15 mmol g^−1^)^28^, and a *badm*Am-based cross-linked polyalkylene (2.3 mmol g^−1^)^5^, respectively. High conductivities can also be realized with relatively low IECs via methods that likely improve the efficiency of hydroxide conduction. Examples include the use of architecture of aliphatic side chain (45 mS cm^−1^, 30 °C, with 1.0 mmol g^−1^ for a *btm*Am-based hexyl-modified PSf)^13^, saturated aliphatic backbone (48 mS cm^−1^ with 1.5 mmol g^−1^ for a trimethyl ammonium-based polyalkylene)^63^, all-benzene-ring backbone (50 mS cm^−1^ with 1.57 mmol g^−1^ for a *btm*Am-based poly(phenylene)^64^, hydrogen-bonding group (1,2,3-triazole) (62 mS cm^−1^ with 1.8 mmol g^−1^ for a trimethyl ammonium-based triazole-containing PPO)^65^, and block copolymer backbone (80 mS cm^−1^ with 1.93 mmol g^−1^ for a poly(fluorenyl sulfone)-co-PSf)^16^.

In summary, Cp^*^_2_Co^+^ shows improved stability and basicity compared with Cp_2_Co^+^, making Cp^*^_2_Co^+^ an ultra-stable cation for polymer HEMs. We have also incorporated Cp^*^_2_Co^+^ cations to a typical polymer backbone (polysulfone) and the resulting membranes exhibited enhanced (short-term) thermal stability and improved (long-term) alkaline stability. More stable polymer backbones are needed to better match the outstanding stability of Cp^*^_2_Co^+^ cation; and the availability of such a stable organic cation might help to develop the next-generation affordable and durable HEM electrochemical devices.

## Methods

### Computational methods for calculating Δ*H*_f_, *δ*_Co_, and p*K*_a_

Heat of formation in gas phase (Δ*H*_f_) of the cation and partial charge at the cobalt atom (*δ*_Co_) were calculated by the software MOPAC2012 (Stewart Computational Chemistry) through the graphical user interface Vega ZZ (version 3.0.1.22, Drug Design Laboratory at the University of Milan). The keyword CHARGE =1 was used to specify the total charge of the cation, and the keyword PRECISE was used to tighten convergence criteria. Conjugate acid dissociation constants (*K*_a_) of two amine groups (basal and terminal) in hexamethylenediamine connected to polysulfone polymer and a benzyl group were predicted using the p*K*_a_ module of the software MarvinSketch (version 5.12.3, ChemAxon Ltd.) through an empirically parameterized method based on partial charge distribution^66^. One repeat unit of polysulfone with hydrogen atoms as end-groups was used in place of the whole polymer for the p*K*_a_ calculation.

### Experimental method for measuring p*K*_b_

Consider the dissociation equilibrium of a general base: MOH 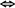 M^+^ + OH^−^. In the equilibrium state, *K*_b_ = ([M^+^]∙[OH^−^])/[MOH], where *K*_b_ is the dissociation constant of the base; [M^+^], [OH^−^], and [MOH] are concentrations of dissociated M^+^, dissociated OH^−^, and undissociated MOH, respectively. When [H^+^] is neglected (reasonable in base), [M^+^] = [OH^−^], so *K*_b_ = [OH^−^]/(*C*_0_/[OH^−^]−1), where *C*_0_ is the initial concentration of MOH. *K*_b_ can be obtained from fitting the slope of the line of [OH^−^] vs. (*C*_0_/[OH^−^]−1). The pH of Cp^*^_2_Co^+^OH^−^ and Cp_2_Co^+^OH^−^ solutions was measured at different initial concentrations (ranging from 0.25 to 50 mM) and the slope was fitted to give *K*_b_. Pure Cp^*^_2_Co^+^OH^−^ and Cp_2_Co^+^OH^−^ were prepared by ion exchange of Cp^*^_2_Co^+^PF_6_^−^ and Cp_2_Co^+^PF_6_^−^, respectively, with a strongly-basic fast-kinetics anion-exchange resin (Amberjet^®^ 4200, Dow Chemical Co.), which had been pre-exchanged with hydroxide in 50 wt% water : 50 wt% methanol, followed by filtration to remove resin and evaporation to remove solvent.

### Synthesis of Br-Cp^*^_2_Co^+^Cl^−^compound

Permethyl-cobaltocenium hexafluorophosphate (Cp^*^_2_Co^+^PF_6_^−^) was brominated by N-bromosuccinimide (NBS) with dibenzoyl peroxide (BPO) as radical initiator and 1,1,2,2-tetrachloroethane (TCE) as solvent. Specifically, 0.474 g (1 mmol) of Cp^*^_2_Co^+^PF_6_^−^ was dissolved in 10 ml TCE, and then 0.178 g (1 mmol) of NBS and 0.0121 g (0.05 mmol) of BPO were added into the solution. After 24 hours of reaction with stirring at 100 °C, brominated permethyl-cobaltocenium hexafluorophosphate (Br-Cp^*^_2_Co^+^PF_6_^−^) was precipitated by pouring the reacted solution into excess diethyl ether. Subsequently, the Br-Cp^*^_2_Co^+^PF_6_^−^ precipitate was filtered, washed thoroughly with diethyl ether, and dried in vacuum at room temperature. The degree of bromination was found to be 95% by NMR spectroscopy (^1^H, Fig. S8 and ^13^C, Fig. S12) and mass spectroscopy (Fig. S13). Second, the Br-Cp^*^_2_Co^+^PF_6_^−^ was ion exchanged with a strongly basic fast-kinetics anion exchange resin balanced with Cl^−^ to produce brominated permethyl-cobaltocenium chloride (Br-Cp^*^_2_Co^+^Cl^−^). Specifically, Br-Cp^*^_2_Co^+^PF_6_^−^ was dissolved in deionized water and then the excess (20 equiv.) anion exchange resin (Amberjet^®^ 4200 with chloride anion) was added into the solution. With stirring, the ion exchange was held at room temperature for 24 hours, and then the resin was removed by filtration. Br-Cp^*^_2_Co^+^Cl^−^ was obtained as a yellow powder by evaporating water from the filtrate. The anion exchange was confirmed by ^19^F NMR spectroscopy with 97% replacement of PF_6_^−^ by Cl^−^ (Fig. S9).

### Synthesis of BAHA-Cp^*^_2_Co^+^Cl^−^ compound

First, benzyl chloride was aminated by excess (20 equiv.) hexamethylenediamine (HMDA). Specifically, 0.127 g (1 mmol) benzyl chloride was dissolved in 10 ml N-methyl-2-pyrrolidone (NMP), then 2.32 g (20 mmol) HMDA and 0.652 g (2 mmol) cesium carbonate were added into the solution. The amination reaction was carried out for 24 hours with stirring at room temperature, then the solvent was removed by adding excess diethyl ether, leaving behind the mixture of desired product (i.e., N-benzylhexane-1,6-diamine, BHDA) and residual HMDA. The HMDA was removed by addition of excess saturated K_2_CO_3_ aqueous solution. The leftover BHDA white power was thoroughly washed with deionized water, followed by drying at 40 °C for 48 hours under vacuum. The synthesis was confirmed by ^1^H NMR spectroscopy with complete mono-amination (Fig. S10). Second, (6-(benzyl amino)hexylamino permethyl-cobaltocenium chloride (BAHA-Cp^*^_2_Co^+^Cl^−^) was synthesized by reacting BHDA with Br-Cp^*^_2_Co^+^Cl^−^. Specifically, 0.206 g (1 mmol) BHDA was dissolved in 10 ml NMP, and 0.444 g (1 mmol) Br-Cp^*^_2_Co^+^Cl^−^ was added into the solution. The reaction was carried out at 80 °C for 24 hours with stirring, then the solvent was removed by adding excess diethyl ether and the BAHA-Cp^*^_2_Co^+^Cl^−^ was obtained after washing by saturated K_2_CO_3_ aqueous solution and deionized water sequentially. The reaction was confirmed by ^1^H NMR spectroscopy with complete conversion (Fig. S11) and complete selectivity for the terminal amine over the basal amine.

### Synthesis of Cp^*^_2_Co^+^-PSf polymer

First, chloromethylated polysulfone (CM-PSf) was synthesized with trimethylchlorosilane and paraformaldehyde as co-chloromethylating agent and stannic chloride as catalyst as reported in detail in our previous work^32^. Second, CM-PSf was aminated with excess HMDA similar to the case of benzyl chloride. Specifically, 0.502 g (1 mmol polysulfone repeat unit or 1.23 mmol chloromethyl group for 123% of DC) CM-PSf was dissolved in 10 ml NMP, then 2.85 g (24.6 mmol) HMDA and 0.802 g (2.46 mmol) cesium carbonate were added into the solution. The amination reaction was held at room temperature for 24 hours with stirring, then the HMDA-aminated polysulfone (HMDA-PSf) white powder was precipitated by pouring the reacted mixture into excess deionized water. After filtration and thorough washing with deionized water, the HMDA-PSf was dried at 40 °C for 48 hours under vacuum. The synthesized HMDA-PSf is completely soluble in organic solvents such as chloroform and NMP, and neither crosslinking nor depolymerization was found for the HMDA-PSf. The synthesis was confirmed by ^1^H NMR spectroscopy with 96% conversion of the chloromethyl groups (Fig. S14).

Second, Cp^*^_2_Co^+^-PSf was synthesized by reacting HMDA-PSf with Br-Cp^*^_2_Co^+^Cl^−^. Specifically, 0.600 g (1 mmol polysulfone repeat unit or 1.23 mmol terminal amine group) HMDA-PSf was dissolved in 10 ml NMP, then 0.546 g (1.23 mmol) Br-Cp^*^_2_Co^+^Cl^−^ was added into the solution. The reaction was completed by heating the mixture at 80 °C for 24 hours with stirring. The reacted solution was cast onto a glass plate and the Cp^*^_2_Co^+^-PSf membranes were obtained after drying at 60 °C for 72 hours. The balancing anion of Cp^*^_2_Co^+^-PSf membranes was exchanged with hydroxide by treatment in 1 M KOH at room temperature for 48 hours. After thorough washing and immersion in deionized water for another 48 hours to completely remove residual KOH, the membranes were ready for use. The synthesis of Cp^*^_2_Co^+^-PSf was confirmed by solid-state ^13^C NMR spectroscopy (Fig. S15).

## Additional Information

**How to cite this article**: Gu, S. *et al.* Permethyl Cobaltocenium (Cp*_2_Co^+^) as an Ultra-Stable Cation for Polymer Hydroxide Exchange Membranes. *Sci. Rep.* **5**, 11668; doi: 10.1038/srep11668 (2015).

## Supplementary Material

Supplementary Information

## Figures and Tables

**Figure 1:**
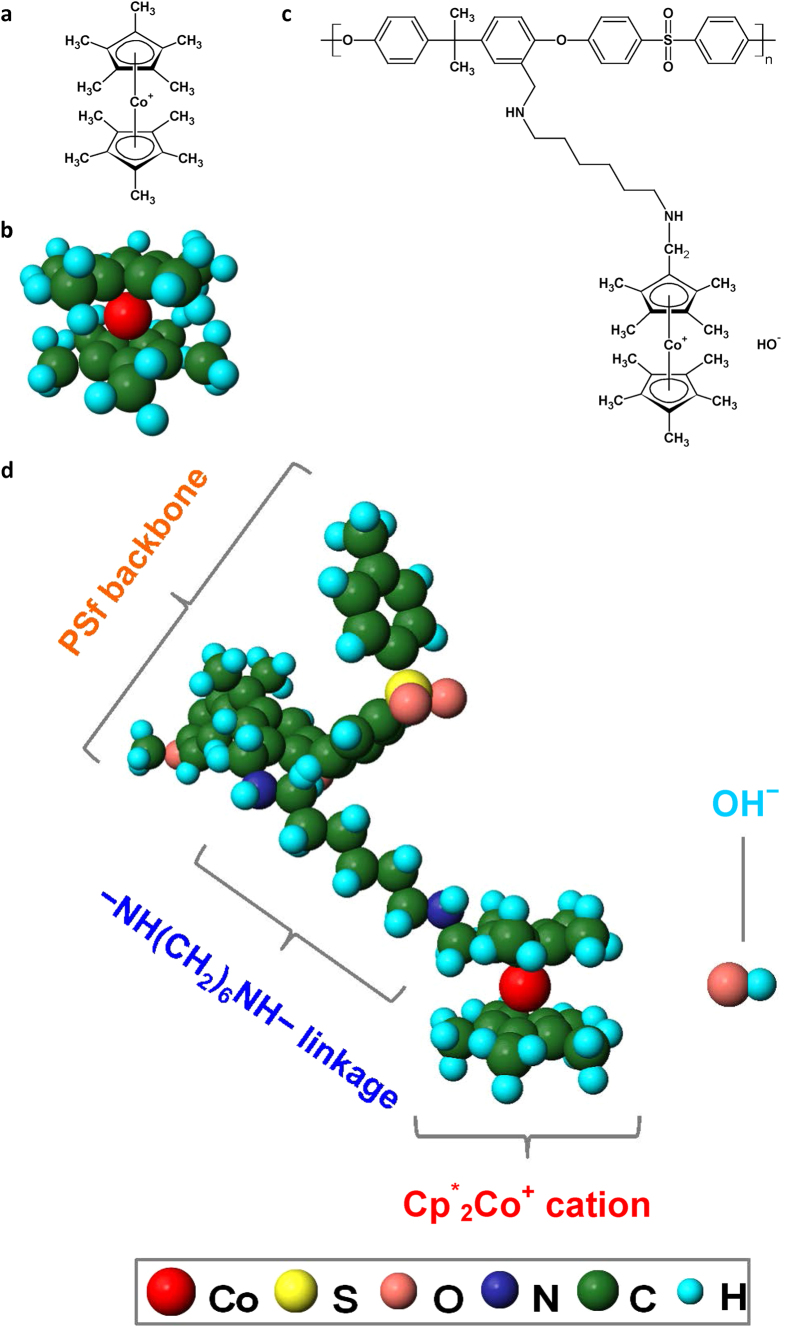
Structure of Cp^*^_2_Co^+^ cation and Cp^*^_2_Co^+^-functionalized polysulfone (Cp^*^_2_Co^+^-PSf). (**a**) Chemical structure of Cp^*^_2_Co^+^. (**b**) Molecular structure of Cp^*^_2_Co^+^. (**c**) Chemical structure of Cp^*^_2_Co^+^-PSf. (**d**) Molecular structure of Cp^*^_2_Co^+^-PSf (one repeat unit of polysulfone shown, predicted by the software MOPAC and drawn in Jmol, version 13.0).

**Table 1:** **Comparison between Cp** ^*^_2_ **Co** ^**+**^ **and Cp**_**2**_ **Co** ^**+**^.

**Cation**	**Δ*****H***_**f**_^a^ **(kJ mol**^**−1**^**, cation)**	***δ***_**Co**_^b^ (***e*****, or 1.602 × 10**^**−19**^ **C)**	***ϕ***^c^ **(V** ***vs.*** **SHE)**	***θ***^d^ **(degree)**	**p*****K***_**b**_^e^ **(for cation hydroxide)**
Cp^*^_2_Co^+^	499	+0.988	−1.24^43^	40.3–49.6	2.7
Cp_2_Co^+^	775^f^	+1.058	−0.63^43^	72.4	5.4

^a^Δ*H*_f_: heat of formation, predicted by the software MOPAC2012 (Stewart Computational Chemistry).

^b^*δ*_Co_: partial charge at the cobalt atom, predicated by the same software.

^c^*ϕ*: formal reductive potential of cation in CH_2_Cl_2_.

^d^*θ*: accessible angle formed by cobalt and the edges of the circumcircles of hydrogen atoms in cation (Fig. S1).

^e^*K*_b_: base dissociation constant for cation hydroxide (Fig. S6).

^f^This calculated value is close to the measured value reported in literature (823 kJ mol^−1^ 68).

**Table 2:** **Membrane properties of different cation-based HEMs (polysulfone backbone when available, chemical structures of cations shown in** **Table S3**).

**Cation**	**HEM**^a^	**IEC**^b^ **(mmol/g)**	**HC**^c^ **(mS/cm)**	**HC**_**IEC**_^d^ **(mS∙g/cm∙mmol)**	**WU**^e^ **(%)**	***T***_**OD**_^f^ **(°C)**	**Ref.**
Cobaltocenium	Cp^*^_2_Co^+^-PSf123	1.20	22	18	68	305	This work
	Cp^*^_2_Co^+^-PSff100	1.09	10	9.2	41		This work
Ammonium	*btm*Am-PSf58, 64^g^	1.08, 1.18	11, 19	10, 19	150, 240	150 (air)	2,46
	*bte*Am-PEK^h^	N/A	14	—	17	200	69
	*ptm*Am-PSf70, 100^i^	1.01, 1.32	15, 26	15, 20	8.7, 12	N/A	70
	*badm*Am-PPO20, 30^j^	1.08, 1.48	7, 14	6.5, 9.5	8.2, 14	N/A	71
	*dbdm*Am-PSf^11^	1.21	11	8.8	N/A	175	72
	*bdabco*Am-PSf^l^	N/A	22	—	N/A	N/A	73
	*bdmp*Am-PSf67^m^	1.23	11 (50 °C)	7.3	50	N/A	74
	*pdm*Am-PDDA39^n^	1.24	0.19	0.15	21	285 (Cl^−^)	75
	*mam*Am-PBI50, 84^o^	1.15, 1.68	0.6, 2.9	0.52, 1.7	28, 50	160	76
	*btam*Am-PSt^p^	N/A	21	—	21	150	49
Imidazolium	*bm*Im-PSf66, 80^q^	1.28, 1.39	9, 16	7.0, 11	25, 8.5	140, 258	77,78
	*am*Im-PStAE20, 30^r^	0.80, 1.20	25, 31	31, 26	87, 116	210	79
	*bdm*Im-PETFE^s^	1.70	17 (HCO_3_^−^)	10	32 (Cl^−^)	N/A	80
	*adm*Im-PFS200^t^	1.04	24	23	17	220	56
	*aam*Im-PStAE60, 80^u^	1.12, 1.50	5, 12	4.5, 8	41, 65	220	57
	*btmtmop*Im-PPO39^v^	1.36	34	25	125	N/A	81
	*dmp*BIm-PBI^w^	N/A	N/A	—	N/A	230	82
	*dmtmp*BIm-PBI64, 80^x^	1.00, 1.50	13.2, 10.1	13.2, 6.7	82, 119	N/A	6
Guanidinium	*bpm*Gu-bpPSf40, 60^y^	0.86, 1.21	5, 12	5.8, 9.9	12, 17	165	28
	*apm*Gu-PPS100^z^	1.39	22	16	32	200	55
	*ppm*Gu-fPSf85^aa^	1.03	21	20	10	N/A	29
Pyridinium	*a*Py-PVPSt50^ab^	1.76	0.6	0.76	30	230 (Br^−^)	27
	*b*Py-PSt34^ac^	1.29	14	11	N/A	130 (DSC)	83
Phosphonium	*bttmop*Ph-PSf124, 152^ad^	1.09, 1.17	27, 45	25, 38	70, 137	187	3,32,48
	*tkdaa*Ph-PCoe17^ae^	0.93	22	24	52	N/A	8
Sulfonium	*mopdp*Su-PSf46^af^	0.69	15	22	27	242	31
Ruthenium	*ttp*Ru-PN9, 17^ag^	1.00, 1.40	14, 29	14, 21	30, 126	N/A	7

^a^The number at the end of the name indicates degree of functionalization.

^b^Ion exchange capacity based on theoretical calculation.

^c^Hydroxide conductivity in deionized water at room temperature.

^d^IEC-normalized hydroxide conductivity.

^e^Water uptake.

^f^Onset decomposition temperature from TGA test (N_2_ atmosphere, 10 °C min^−1^).

^g^Benzyl-trimethylammonium (*btm*Am).

^h^Benzyl-triethylammonium (*bte*Am), poly(ether ketone) (PEK).

^i^Phenyl-trimethylammonium (*ptm*Am).

^j^Benzyl-alkyl-dimethylammonium (*badm*Am), poly(phenylene oxide) (PPO).

^k^Dibenzyl-dimethylammonium (*dbdm*Am).

^l^Benzyl-1,4-diazabicyclo-[2.2.2]-octane-ammonium (*bdabco*Am).

^m^Benzyl-(1,4-dimethyl)piperazine-ammonium (*bdmp*Am).

^n^Pyrrolidine-dimethylammonium (*pdm*Am), poly(diallyldimethylammonium) (PDDA).

^o^Morpholine-alkyl-methylammonium (*mam*Am), polybenzimidazole (PBI).

^p^Benzyl-1,3,5-triazine-methylammonium (*btam*Am), polystyrene (PSt).

^q^Benzyl-1-methyl-imidazolium (*bm*Im).

^r^Alkyl-1-methyl-imidazolium (*am*Im), crosslinked poly(styrene acrylonitrile ethylene) (PStAE).

^s^Benzyl-1,2-dimethyl-imidazolium (*bdm*Im), grafted poly(ethylene tetrafluoroethylene) (PETFE) backbone.

^t^Alkyl-1,2-dimethyl-imidazolium (*adm*Im), poly(fluorene sulfone) (PFS).

^u^Alkyl-1-alkyl-2-methyl-imidazolium (*aam*Im).

^v^Benzyl-1,4,5-trimethyl-2-(2,4,6-trimethoxyphenyl)-limidazolium (*btmtmop*Im).

^w^1,3-Dimethyl-2-phenyl-benzimidazolium (*dmp*BIm).

^x^1,3-Dimethyl-2-(2,4,6-Trimethylphenyl)-benzimidazolium (*dmtmp*BIm).

^y^Benzyl-pentamethylguanidinium (*bpm*Gu), biphenylene-type polysulfone (bpPSf).

^z^Alkyl-pentamethylguanidinium (*apm*Gu), poly(phenolphthalein sulfone) (PPS).

^aa^Phenyl-pentamethylguanidinium (*ppm*Gu), fluorinated polysulfone (fPSf).

^ab^Alkylpyridinium (*a*Py), poly(vinylpyridine-styrene) (PVPSt).

^ac^Benzylpyridinium (*b*Py), crosslinked backbone.

^ad^Benzyl-tris(2,4,6-trimethoxyphenyl)-phosphonium (*bttmop*Ph).

^ae^Tetrakis(dialkylamino)phosphonium (*tkdaa*Ph), polycyclooctene (PCoe).

^af^(4-Methoxyphenyl)-diphenylsulfonium (*mopdp*Su).

^ag^Bis(terpyridinine)ruthenium (*btp*Ru), polynorbornene (PN).
