# Supplementary material for: Permethyl Cobaltocenium (Cp*2Co+) as an Ultra-Stable Cation for Polymer Hydroxide-Exchange Membranes
Source: Sci Rep. 2015 Jun 29;5:11668. doi: 10.1038/srep11668 (PMC4484364; doi:10.1038/srep11668)
Supplement: Supplementary Information [file srep11668-s1.pdf]

Supplementary Information for

Permethyl Cobaltocenium ($Cp^*_2Co^+$) as an Ultra-Stable Cation for Polymer Hydroxide-Exchange Membranes

Shuang Gu[1], Junhua Wang[1], Robert B. Kaspar[1], Qianrong Fang[1], Bingzi Zhang[1], E. Bryan Coughlin[2] & Yushan Yan[1,*]

[1] Department of Chemical & Biomolecular Engineering, Center for Catalytic Science and Technology, University of Delaware, Newark, Delaware 19716, USA.

[2] Department of Polymer Science and Engineering, University of Massachusetts, Amherst, Massachusetts 01003, USA.

Correspondence and requests for materials should be addressed to Y.S.Y.
(email: yanys@udel.edu)

1. Supplementary Methods

Characterization of cation stability. Alkaline stability was evaluated by monitoring ^{1}H NMR spectroscopy over time (up to 1,000 hours) in a deuterated alkaline solution (1 M NaOD in D_2O) using a hydrothermal method. For example, 10 mg of $Cp^*_2Co^+OH^-$ was dissolved in 2.0 ml 1 M NaOD in D_2O in a thick-walled glass tube, which was then flash-frozen in dry ice, evacuated, flame-sealed, buried in sand in a metal container, and then transferred to an oven. Slight glass dissolution was observed during the test but it did not compromise NaOD or D_2O in the test solution. Caution: high pressure (3.6 atm est.) may be built up in the tube during the test.

Characterization of membrane properties. Hydroxide conductivity was measured at room temperature (20 °C) in deionized water with a four-electrode test apparatus (Pt wire electrodes) using an AC impedance analyzer and a potentiostat (frequency range: 1–10^5 Hz). To avoid CO_2 contamination from ambient air, pure nitrogen was flowed through the system at a high rate. For measuring the conductivity at elevated temperatures (*e.g.*, 60 °C or 80 °C), a glass container with gas atmosphere control filled with a sufficient amount of deionized water was fixed into the oil bath, and the four-electrode test apparatus was completely immersed into the deionized water of the container and the temperature was controlled by the oil bath. Water uptake was obtained by comparing wet and dry membrane weights. Detailed information about hydroxide conductivity and water uptake measurements were reported in our previous work(1, 2). IEC was measured by back-titration method. $Cp^*_2Co^+$-PSf membranes with OH^- form were immersed and neutralized by excess 0.1 M HCl solution, and then the resulting solution was titrated by 0.1 M NaOH solution. The IEC was calculated by the following equation [IEC = $(C_{HCl}\cdot V_{HCl} - C_{NaOH}\cdot V_{NaOH})/(3m)$, where C_{HCl} and C_{NaOH} are molar concentration of HCl solution and NaOH one, respectively; V_{HCl} and V_{NaOH} are the used volume of HCl solution and the titrated volume of NaOH solution, respectively; and m is the weight of dry membrane sample. The value of "3" here is because two amine groups also contribute to the IEC measurement along with one cobaltocenium group]. For tests

of hydroxide conductivity, water uptake, and IEC, each sample consisted of three replicates (0.5 g per replicate) and the average of the three replicates was reported. The uncertainty was around 5% for IEC and around 10% for both hydroxide conductivity and water uptake. Transmission electron microscopy (TEM) images of membranes were taken on JEM-2010F, JOEL. An ultra-thin membrane sample was prepared by drop casting onto a copper grid, followed by drying at 80 ˚C for 4 hours. Prior to imaging, the membrane was washed in deionized water and dried for 12 hours at room temperature. Tensile strength of 3 cm x 0.5 cm samples was evaluated with a mechanical analyzer (Model 1211, Instron) at a cross-head speed of 10 mm/min at room temperature.

Characterization of membrane stability. Thermogravimetric analysis (TGA) was conducted using a thermogravimetric/differential thermal analyzer (TGA/DSC 1, Mettler Toledo) under nitrogen with a flow rate of 100 ml/min. The sample was held at 100 °C for 10 min to completely remove water and then heated to 700 °C at a ramp rate of 10 ˚C/min. A derivative thermogravimetric (DTG) curve was obtained from the first derivative of the TGA curve with respect to temperature. Alkaline stability was evaluated by monitoring ion-exchange capacity over time (up to 2,000 hours) in alkaline solutions (1 M KOH at 80 ˚C or 100 ˚C). Each sample consisted of three replicates (0.5 g per replicate) and the average of the three replicates was reported. Specifically, 0.5 g membrane sample was completely immersed into 100 ml alkaline solution in a plastic bottle that was tightly-sealed to prevent water evaporation or CO_2 contamination. The plastic bottle was buried in sand in a metal container to maintain uniform temperature and to avoid isolation from environmental fluctuations, and then the metal container was placed in an oven. Caution: high pressure (1 atm est.) may be built up in the vial during the test at 100 ˚C.

2. Supplementary Tables (S1–S3)

Table S1. Measured pH of aqueous solutions of $Cp^*_2Co^+OH^-$ and $Cp_2Co^+OH^-$. See **Fig. S6** for the base dissociation constants (K_b).

Base	C_0[a] / mmol L^{-1}	pH[b]	$[OH^-]$[c] / mmol L^{-1}
$Cp^*_2Co^+OH^-$	50	11.84	6.92
	25	11.66	4.57
	5	11.06	1.15
	1	10.78	0.603
	0.5	10.21	0.158
$Cp_2Co^+OH^-$	25	10.57	0.372
	12.5	10.37	0.234
	2.5	10.18	0.151
	0.5	10.03	0.107
	0.25	9.64	0.0437

[a] Initial base concentration, where the base solution was prepared by dissolving a certain amount of pure base in deionized water.

[b] Measured with a calibrated pH meter.

[c] Calculated from pH.

Table S2. Calculated acid dissociation constants (K_a) of the conjugate acids of terminal and basal amines.

Molecule	pK_a of amine conjugate acid[a]	
	Terminal amine	Basal amine
HMDA-aminated benzyl chloride	10.21	9.93
HMDA-aminated chloromethylated polysulfone	10.21	9.26

[a] Predicted by the software MarvinSketch (version 2.12.3, ChemAxon Ltd)

Table S3. Chemical structures of reported HEM cations.			
Cation	Chemical structure		
Ammonium	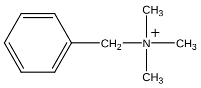<br>Benzyl–trimethylammonium (*btm*Am)	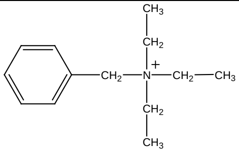<br>Benzyl–triethylammonium (*bte*Am)	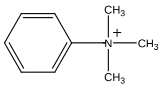<br>Phenyl–trimethylammonium (*ptm*Am)
	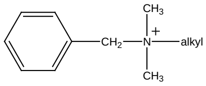<br>Benzyl–alkyl–dimethylammonium (*badm*Am)	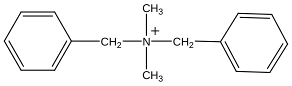<br>Dibenzyl–dimethylammonium (*dbdm*Am)	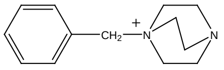<br>Benzyl–1,4–diazabicyclo–[2.2.2.]–octane–ammonium (*bdabco*Am)
	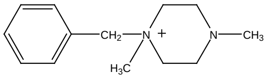<br>Benzyl–(1,4–dimethyl)piperazine–ammonium (*bdmp*Am)	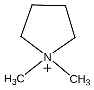<br>Pyrrolidine–dimethtylammonium (*pdm*Am)	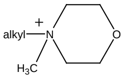<br>Morpholine–alkyl–methylammonium (*mam*Am)
	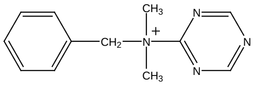<br>Benzyl–1,3,5–triazine–methylammonium (*btam*Am)		
Imidazolium	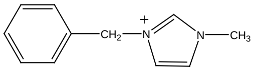<br>Benzyl–1–methyl–imidazolium (*bm*Im)	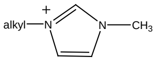<br>Alkyl–1–methyl–immidazolium (*am*Im)	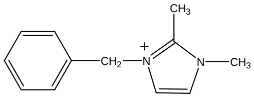<br>Benzyl–1,2–dimethyl–imidazolium (*bdm*Im)
	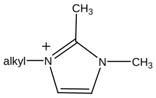<br>Alkyl–1,2–dimethyl–imidazolium (*adm*Im)	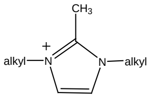<br>Alkyl–1–alkyl–2–methyl–immidazolium (*aam*Im)	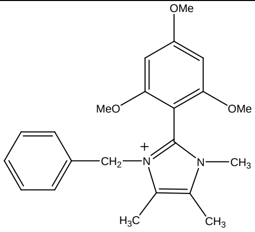<br>Benzyl–1,4,5–trimethyl–2–(2,4,6–trimethoxyphenyl)–immidazolium (*btmtmop*Im)
	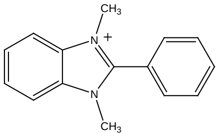<br>1,3–Dimethyl–2–phenyl–benzimidazolium (*dmp*BIm)	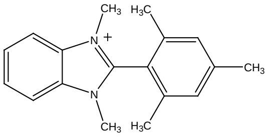<br>1,3–Dimethyl–2–(2,4,6–trimethylphenyl)–benzimidazolium (*dmtmp*BIm)	
Guanidinium	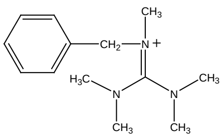<br>Benzyl–pentamethylguanidinium (*bpm*Gu)	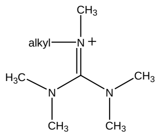<br>Alkyl–pentamethylguanidinium (*apm*Gu)	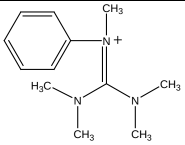<br>Phenyl–pentamethylguanidinium (*ppm*Gu)

8Pyridinium	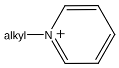 Alkyl−pyridinium (*a*Py) 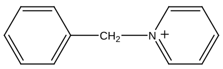 Benzyl−pyridinium (*b*Py)
Phosphonium	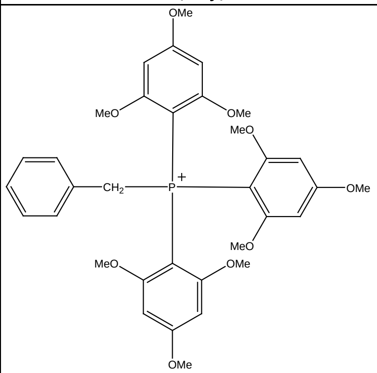 Benzyl−tris(2,4,6−trimethoxyphenyl)−phosphonim (*bttmop*Ph) 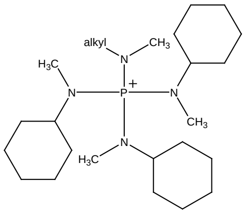 Tetrakis(dialkylamino)phosphonium (*tkdaa*Ph)
Sulfonium	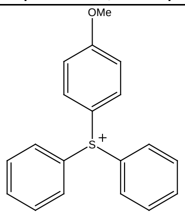 (4−Methoxyphenyl)−diphenylsulfonium(*mpdp*Su)
Ruthenium	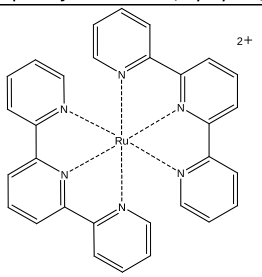 Bis(terpyridinine)ruthenium (*btp*Ru)

3. Supplementary Figures (S1–S21)

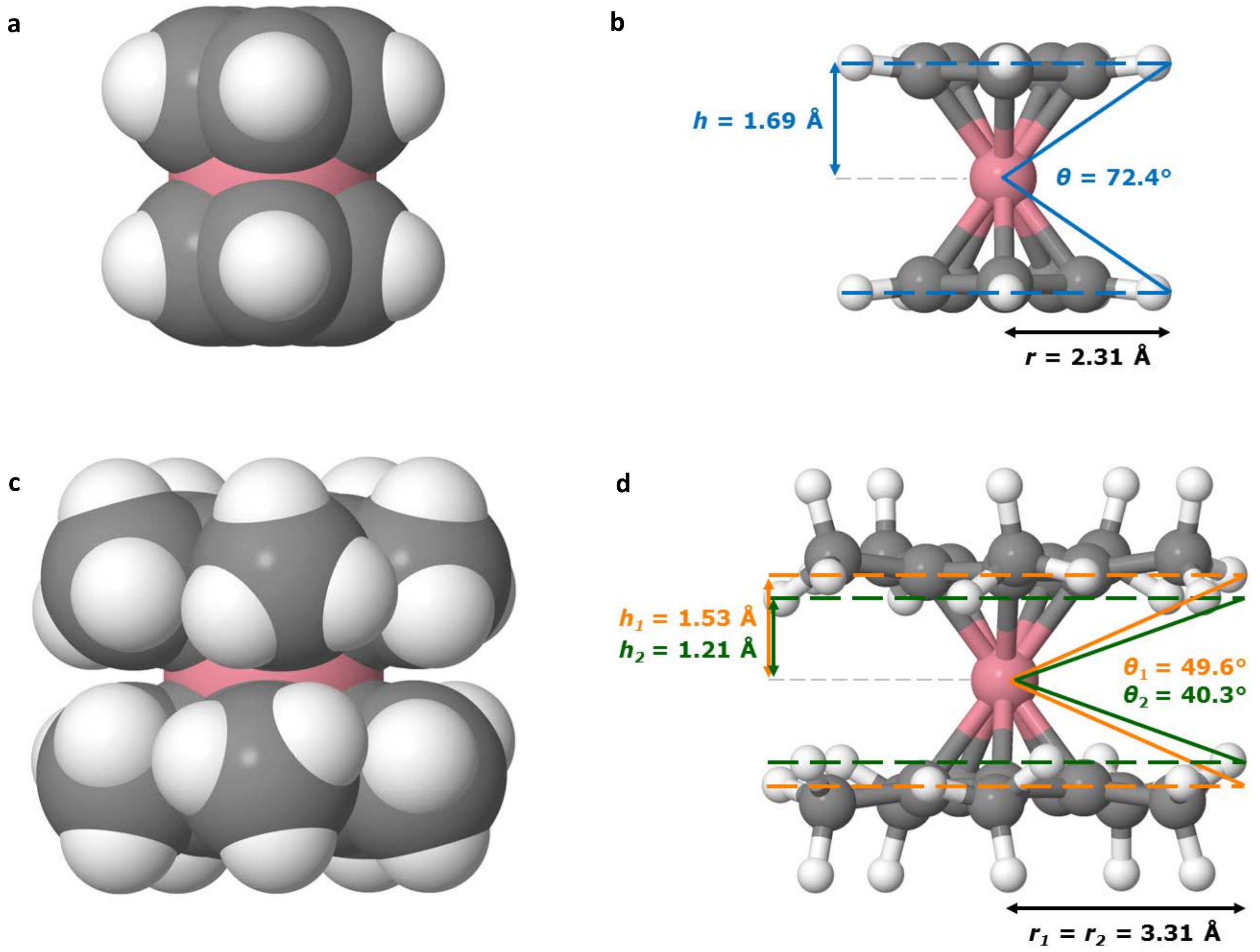

Fig. S1. Steric hindrance of Cp_2Co^+ and $Cp^*_2Co^+$ cations. (**a**) The geometry of Cp_2Co^+ cation with minimum system energy computed in vacuum. (**b**) Accessible angle (θ) in Cp_2Co^+ cation formed by cobalt center and the edges of circumcircles encompassing the hydrogen atoms. The coordinates of the ten hydrogen atoms were used to calculate the radius (r) of the circumcircles of the cyclopentadienyl rings; the height (h) is from cobalt to a circumcircle. (**c**) The geometry of $Cp^*_2Co^+$ cation with minimum system energy computed in vacuum. (**d**) Accessible angle (θ) in $Cp^*_2Co^+$ cation formed by cobalt center and the edges of circumcircles. After the hydrogen atoms were grouped by height into three sets of five, the same calculations were repeated separately for the two groups closest to cobalt.

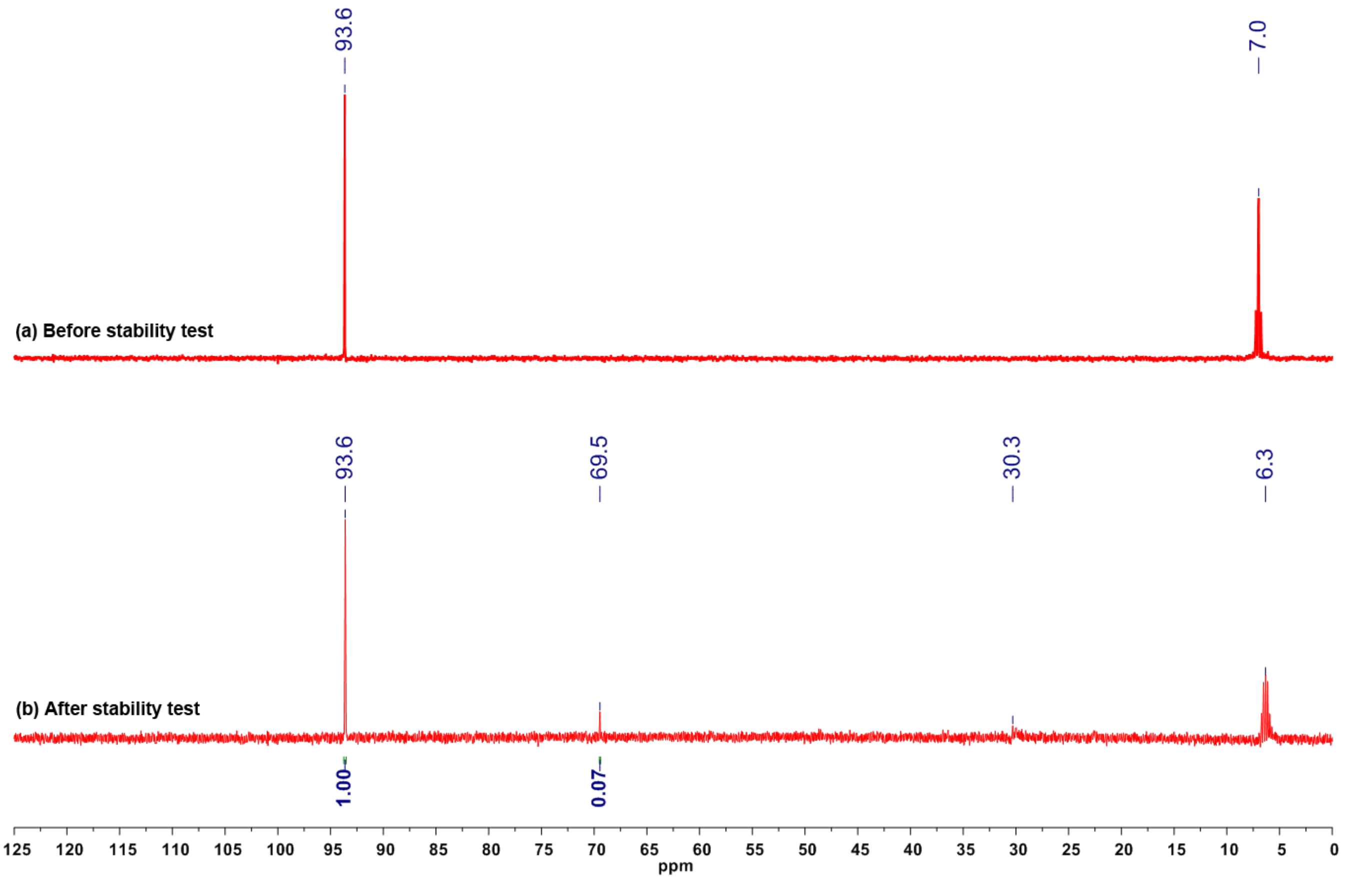

Fig. S2. ^{13}C NMR spectra of $Cp^*_2Co^+OH^-$ subjected to the alkaline stability test (140 °C, 1 M NaOD/D_2O, 6 weeks or 1,000 hours). (**a**) Before test. (**b**) After test. Conditions: 10 mg $Cp^*_2Co^+OH^-$ in 2 mL of 1 M NaOD in D_2O, 140 °C, 1,000 hours. The peaks at 93.6 ppm and 7.0 ppm are from the ring and methyl carbons of $Cp^*_2Co^+$, respectively(3). The slight change in the chemical shift of the methyl carbons from 7.0 ppm to 6.3 ppm is due to complete H-D isotopic exchange during the test (see **Fig. S21**); the ring carbons show no change. Two peaks form during the test at 69.5 ppm and 30.3 ppm, suggesting *tert*-butyl alcohol as the degraded product. However, the detailed degradation mechanism is still not understood for now and will be studied in the future. The degree of degradation (DD) can be calculated by the equation: DD = [(A2+A3)/(A1+A2+A3)] × 100%, where A1, A2, and A3 are the integrals of peaks at 93.6 ppm, 69.5 ppm, and 30.3 ppm, respectively. The results showed that 8.5% of the $Cp^*_2Co^+$ degraded in 1,000 hours.

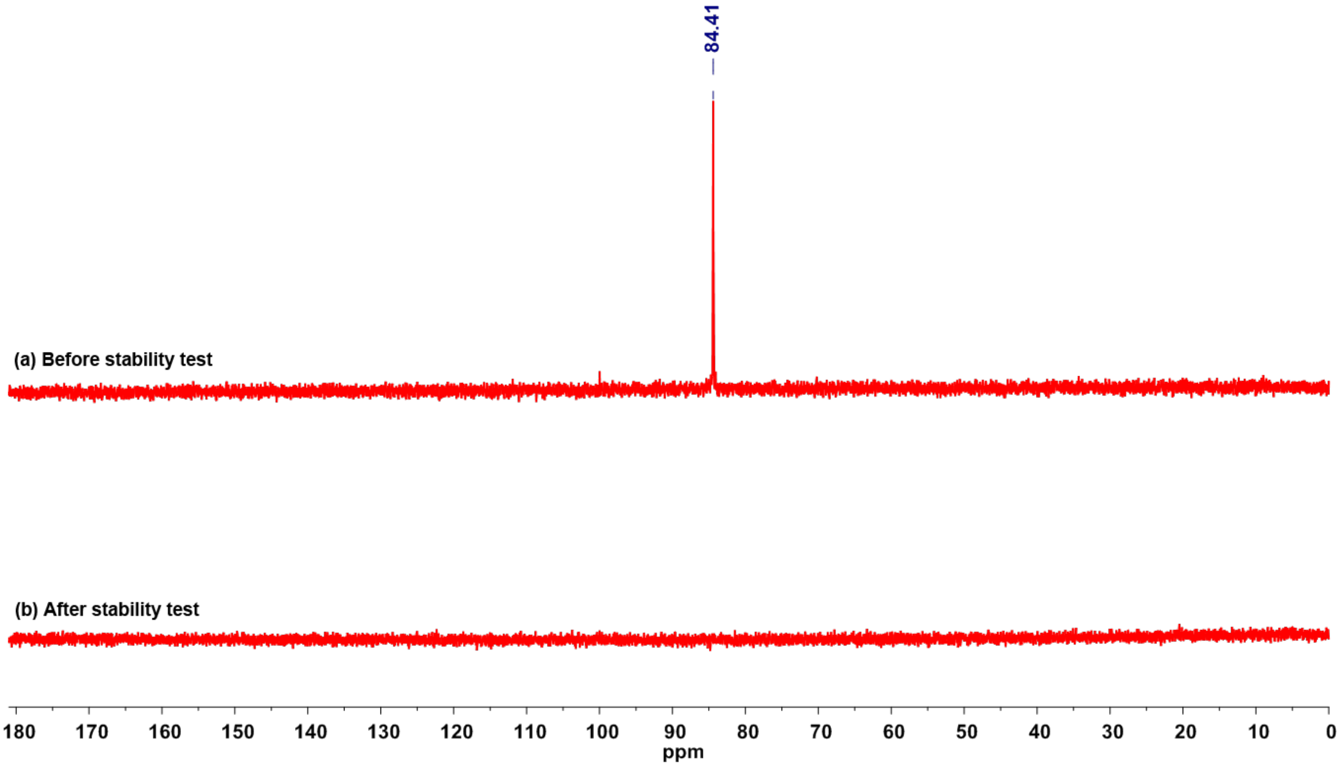

Fig. S3. ^{13}C NMR spectra of $Cp_2Co^+OH^-$ subjected to the alkaline stability test (140 °C, 1 M NaOD/D_2O, 1 week or 168 hours). (**a**) Before test. (**b**) After test. Conditions: 10 mg $Cp_2Co^+OH^-$ in 2 mL of 1 M NaOD in D_2O, 140 °C, 168 hours. The peak at 84.41 ppm is from (ring) carbons of Cp_2Co^+. The results showed that Cp_2Co^+ completely degraded during the stability test; a black precipitate was observed as the degradation product. The NMR spectra were obtained by traditional liquid ^{13}C NMR spectrometer and the carbon signals can be detected only from the solubilized molecules. The disappearance of carbon signal suggests that no soluble carbon-containing specie was formed during the degradation. A black precipitate was observed after the stability test, and thus it is plausible that all carbon elements were in the insoluble precipitate.

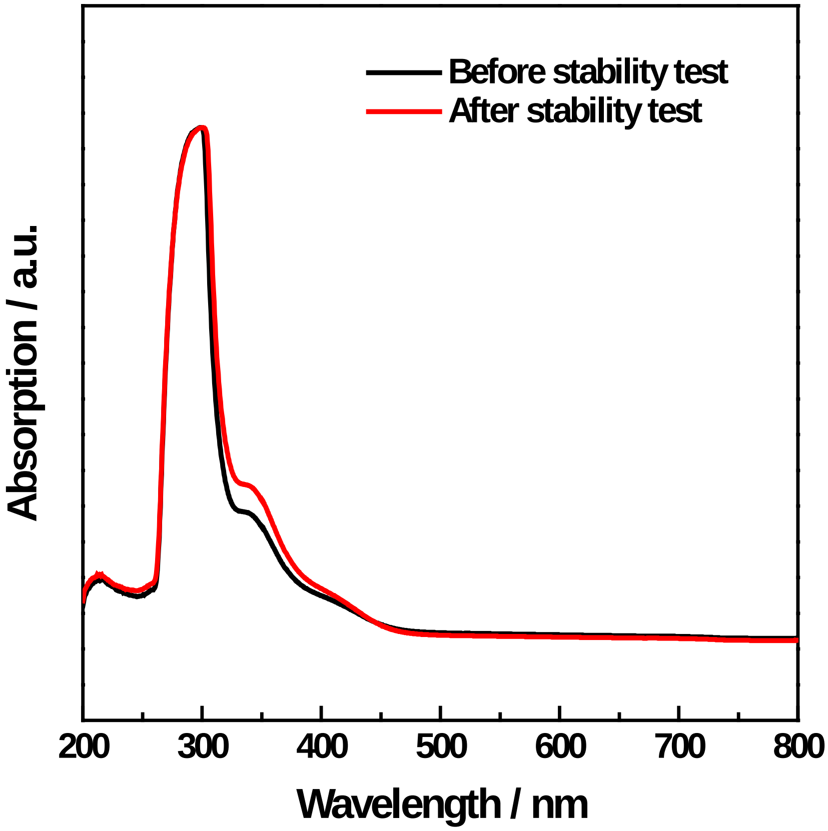

Fig. S4. UV-Vis absorption spectra of $Cp^*_2Co^+OH^-$ subjected to the alkaline stability test (140 °C, 1 M NaOD/D_2O, 6 weeks or 1,000 hours). Before test: black curve; after test: red curve. Conditions: see **Fig. S2**. The concentration of $Cp^*_2Co^+OH^-$ was diluted to 10 ppm before measurement with a UV-Vis spectrophotometer (JASCO V-550, Ubest). Before the test, $Cp^*_2Co^+$ shows absorption peaks at 300 nm (strong), 340 nm (shoulder), and 410 nm (weak bond), which are induced by the ground-excitation transition of π electrons in the aromatic rings.(4) After the test, the positions and relative intensities of these three characteristic peaks remained almost the same, indicating that $Cp^*_2Co^+$ retained integrity, in agreement with the ^{13}C NMR spectroscopy results.

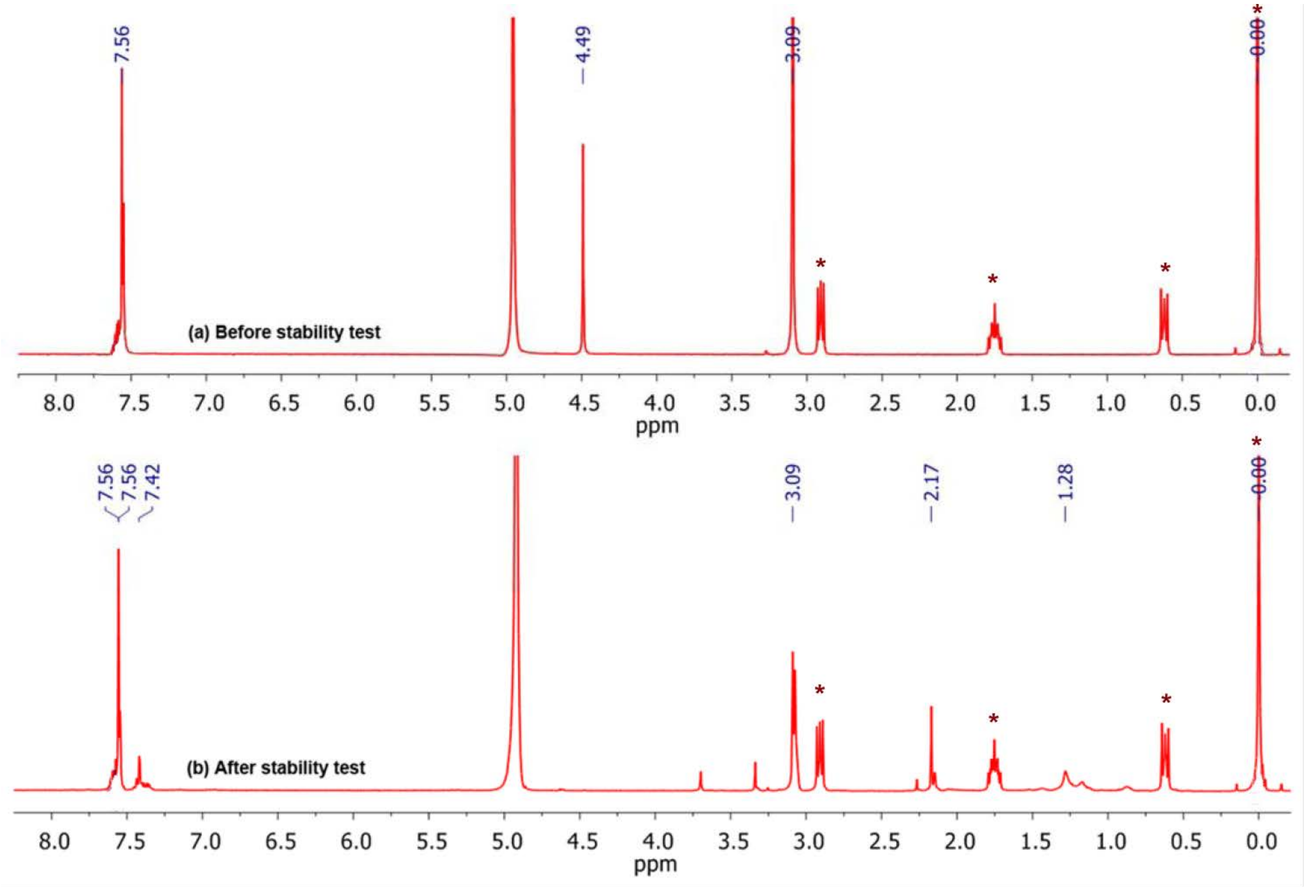

Fig. S5. ^{1}H NMR spectra of trimethyl benzylammonium subjected to the alkaline stability test (140 °C, 1 M KOD/D_2O, 24 hours). (**a**) Before test. (**b**) After test. Conditions: 10 mg trimethyl benzylammonium hydroxide in 2 mL of 1 M KOD in D_2O, 140 °C, 24 hours, 3-(trimethylsilyl)-propane sulfonic sodium as internal standard (2.90, 1.75, 0.60, and 0 ppm, all labeled "*"). Before the test, the cation shows peaks for aromatic (7.56 ppm), methylene (4.49 ppm), and methyl protons (3.09 ppm). After the test, the following degradation products appeared: benzyl dimethylamine (~7.42, 3.3, 2.17 ppm), benzyl alcohol (~7.4 ppm), and trimethylamine (1.28 ppm). Note that other degradation products also appear. Solely considering the two degradation products above, the degree of degradation (DD) can be calculated by the equation: DD = [(3A2+2A3)/2A1] × 100%, where A1 is the integral of methyl protons (3.09 ppm) of trimethyl benzylammonium; and A2 and A3 are the integrals of the methyl protons (2.17 ppm) of benzyl dimethylamine degradation product and methyl protons (1.28 ppm) of trimethylamine degradation product, respectively. The results show a degree of degradation of 18% of the trimethyl benzylammonium degraded in 24 hours.

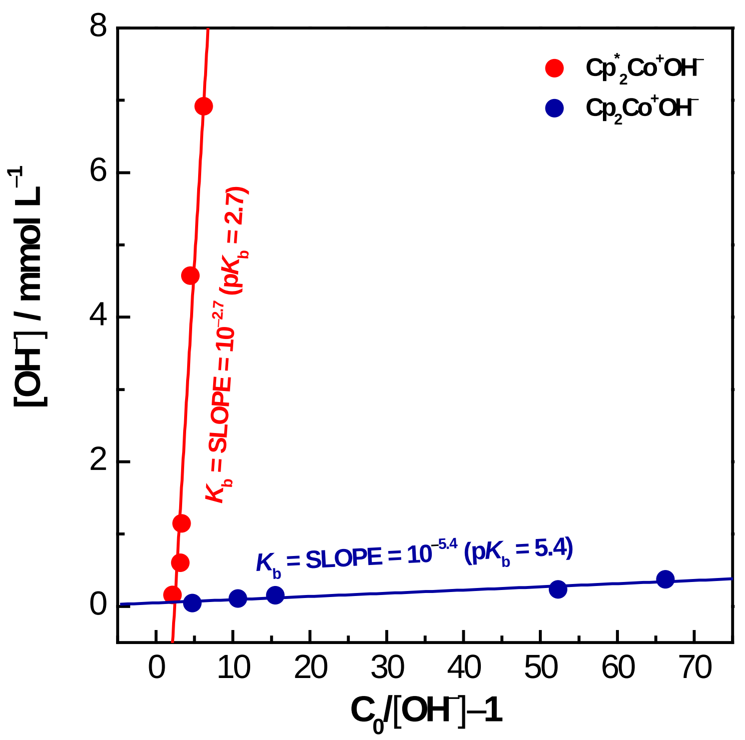

Fig. S6. Measurement of base dissociation constant (K_b) for $Cp^*_2Co^+OH^-$ and $Cp_2Co^+OH^-$. C_0 is the initial base concentration. For the K_b fit, R^2 = 0.93, 0.90 for $Cp^*_2Co^+OH^-$ and $Cp_2Co^+OH^-$, respectively.

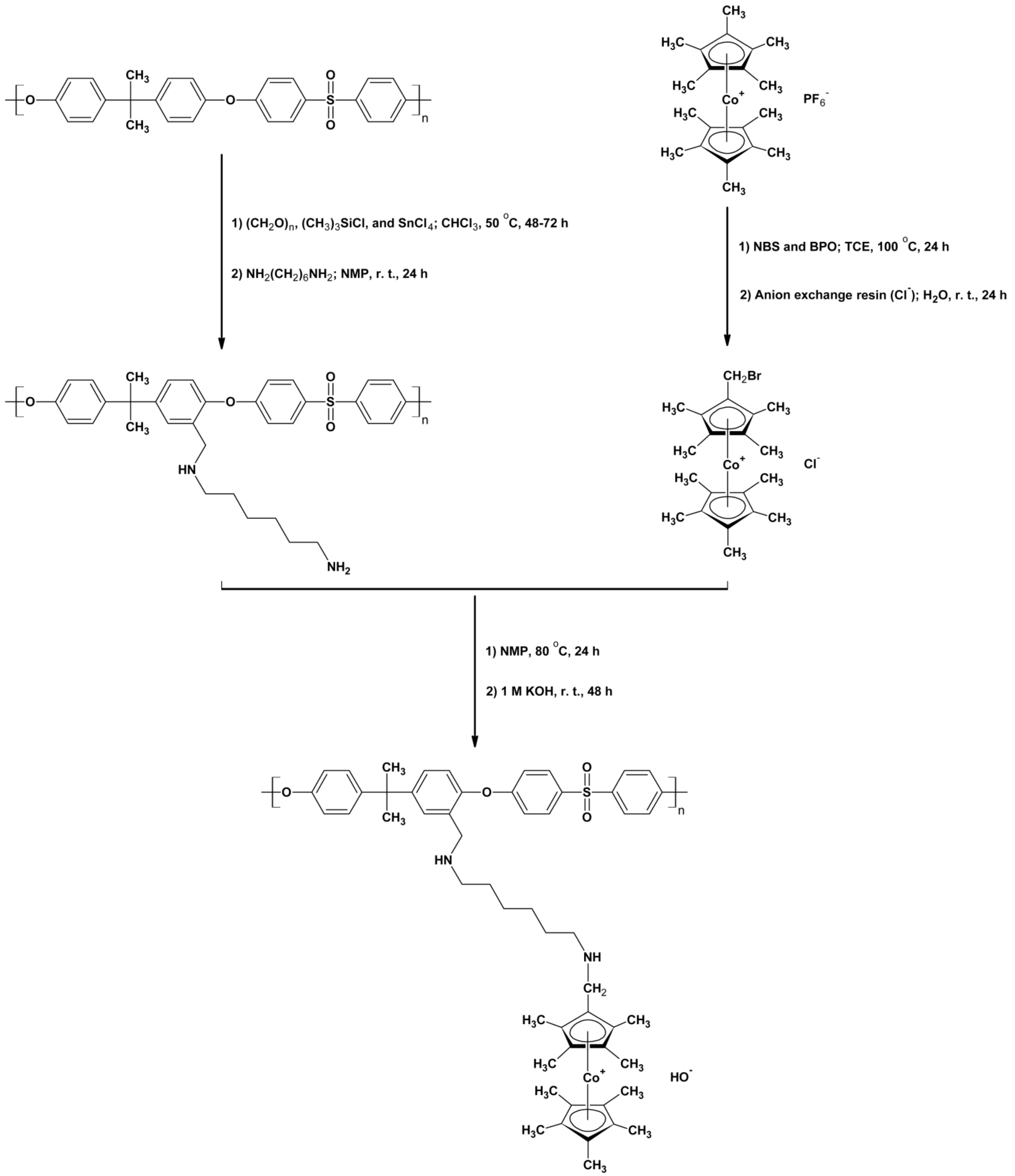

Fig. S7. Synthesis of permethyl-cobaltocenium-functionalized polysulfone ($Cp^*_2Co^+$-PSf).

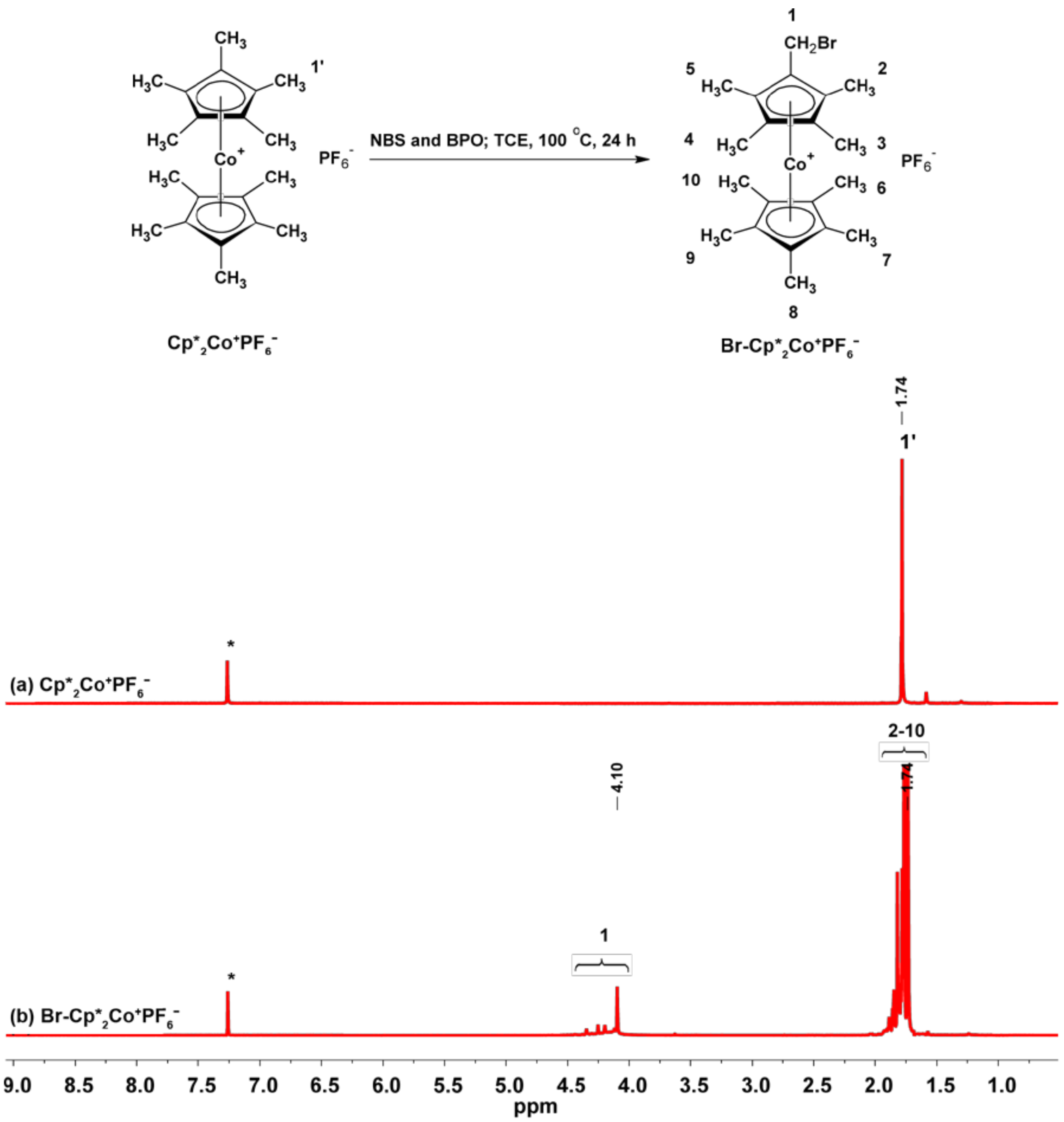

Fig. S8. ^{1}H NMR spectroscopy evidence for bromination of permethyl cobaltocenium hexafluorophosphate ($Cp^*_2Co^+PF_6^-$). (**a**) Before bromination ($Cp^*_2Co^+PF_6^-$). (**b**) After bromination ($Br\text{-}Cp^*_2Co^+PF_6^-$). $CDCl_3$ was used as solvent for both cases (7.26 ppm, labeled "*"). Before bromination, there is only one strong peak at 1.74 ppm (methyl protons). After bromination, a new peak appears at 4.10 ppm (bromomethyl protons). Note that a few small peaks near 4.10 ppm are also from bromomethyl protons; this change in chemical shift is likely caused by ring rotation(5). These results are consistent with the ^{13}C NMR spectroscopy results (**Fig. S12**). The degree of bromination (DB) can be calculated from the $Br\text{-}Cp^*_2Co^+PF_6^-$ spectrum by the equation: DB = [30A1/(3A1+2A2)] × 100%, where A1 and A2 are the integral of peak 1 and the sum of the integrals of peaks 2−10, respectively.

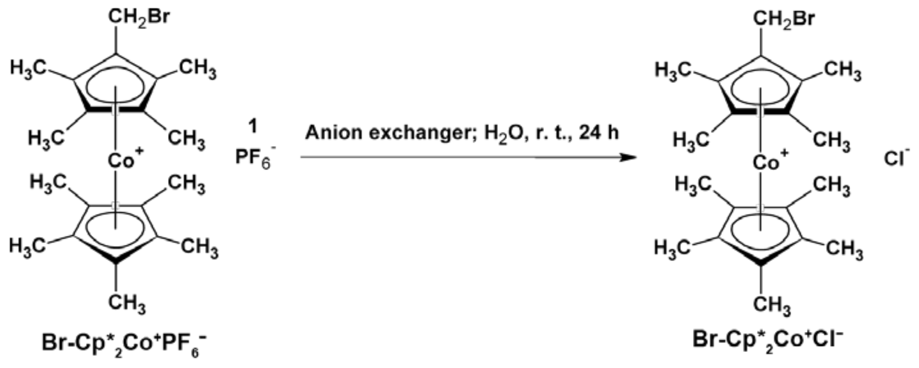

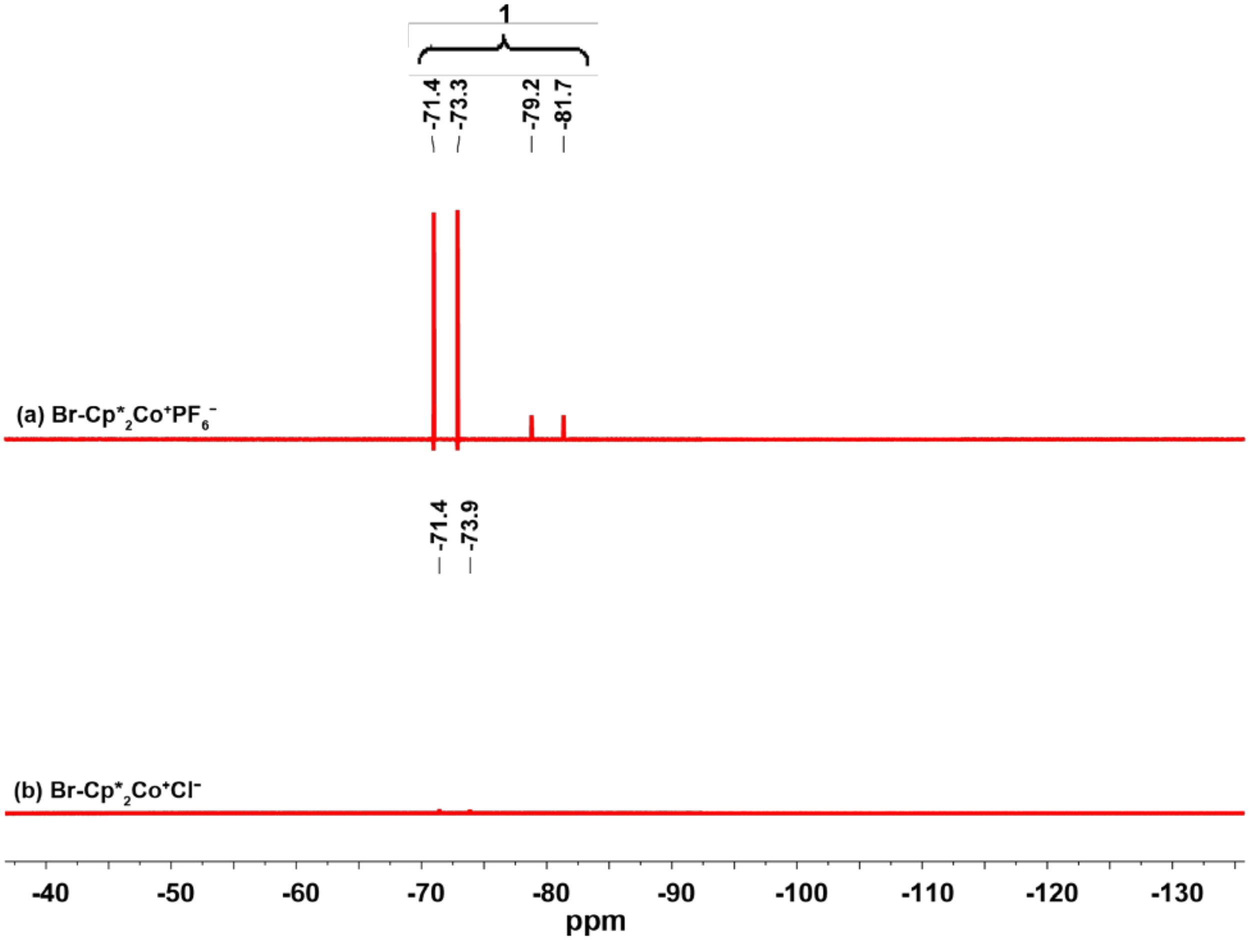

Fig. S9. ^{19}F NMR spectroscopy evidence for anion exchange of brominated permethyl cobaltocenium hexafluorophosphate ($Br\text{-}Cp^*_2Co^+PF_6^-$). (**a**) Before ion exchange ($Br\text{-}Cp^*_2Co^+PF_6^-$). (**b**) After ion exchange ($Br\text{-}Cp^*_2Co^+Cl^-$). DMSO-d_6 was used as solvent for both cases. $Br\text{-}Cp^*_2Co^+PF_6^-$ shows twin peaks at 71.4 and 73.3 ppm for the PF_6^- anion, consistent with literature.(6, 7) The adjacent twin peaks at 79.2 and 81.7 ppm are likely caused by ring rotation (as with the ^{1}H NMR spectrum). After anion exchange, only traces of the original peaks remain, suggesting a degree of anion exchange of about 97%.

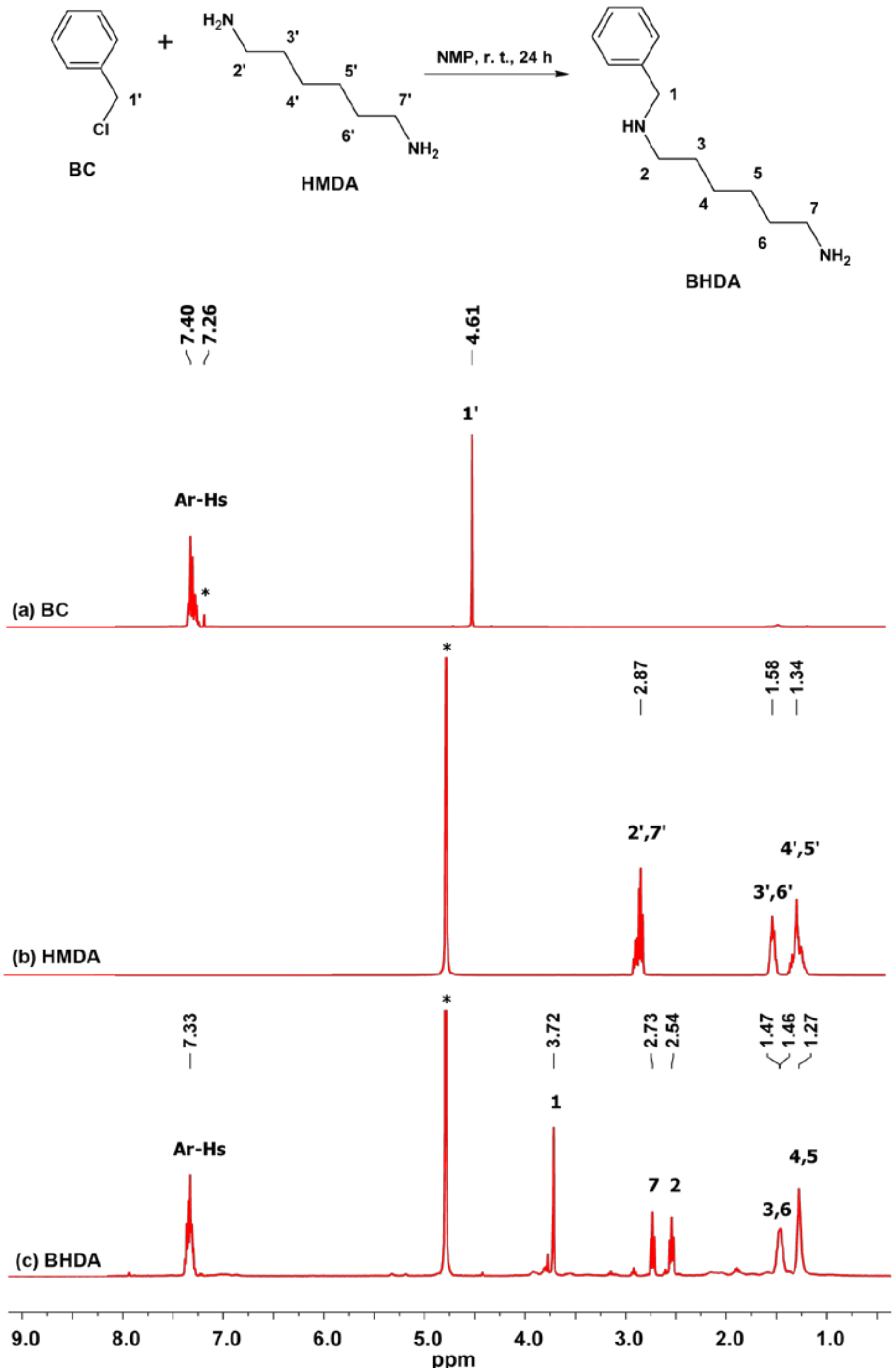

Fig. S10. ^{1}H NMR spectroscopy evidence for synthesis of N-benzylhexane-1,6-diamine (BHDA). (**a**) Benzyl chloride (BC) reactant. (**b**) Hexamethylenediamine (HMDA) reactant. (**c**) N-benzylhexane-1,6-diamine (BHDA) product. $CDCl_3$ was used as solvent (7.26 ppm, labeled "*") for BC, and D_2O was used as solvent (4.79 ppm, labeled "*") for HMDA and BHDA. The degree of amination (DA) can be calculated from the equation: DA = (2A1/A2) ×100%, where A1 and A2 are the integral of peak 1 and the sum of the integrals of peaks 2 and 7, respectively.

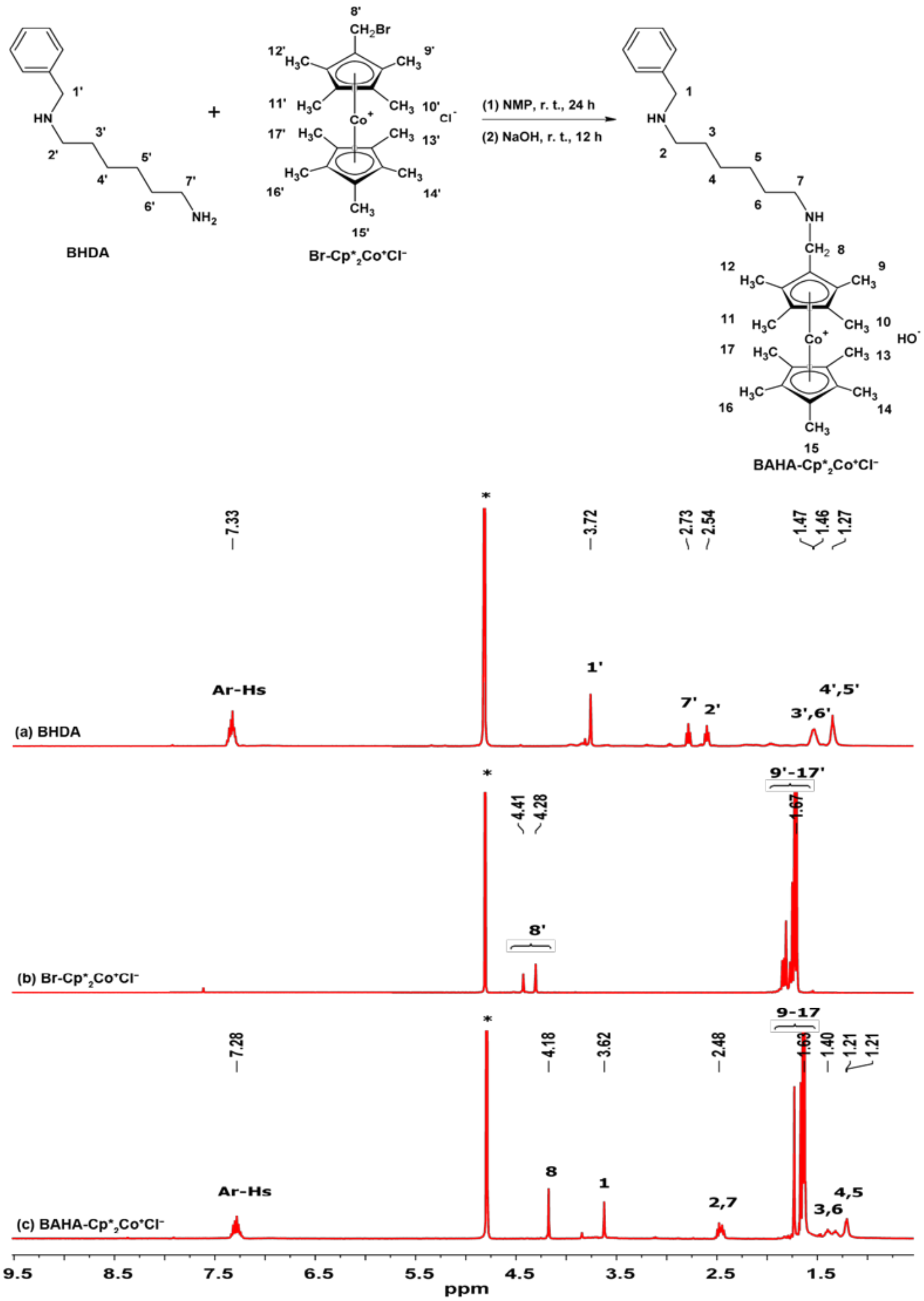

Fig. S11. ^{1}H NMR spectroscopy evidence for synthesis of (6-benzyl amino)hexylamino permethyl cobaltocenium hydroxide (BAHA-Cp$^*_2$Co$^+$OH$^-$). (**a**) N-benzylhexane-1,6-diamine (BHDA). (**b**) Brominated permethyl-cobaltocenium chloride (Br-Cp$^*_2$Co$^+$Cl$^-$). (**c**) (6-Benzyl amino)hexylamino permethyl cobaltocenium hydroxide (BAHA-Cp$^*_2$Co$^+$OH$^-$). D_2O was used as solvent (4.79 ppm, labeled “*”). The degree of cobaltocenium functionalization (DCF) can be calculated from the BAHA-Cp$^*_2$Co$^+$OH$^-$ spectrum by the equation: DCF = (A1/A2) × 100%, where A1 and A2 are the integrals of peaks 8 and 1, respectively. Disappearance of the peak numbered “7” (2.73 ppm) in the product spectrum confirmed complete selectivity for the terminal amine over the basal one.

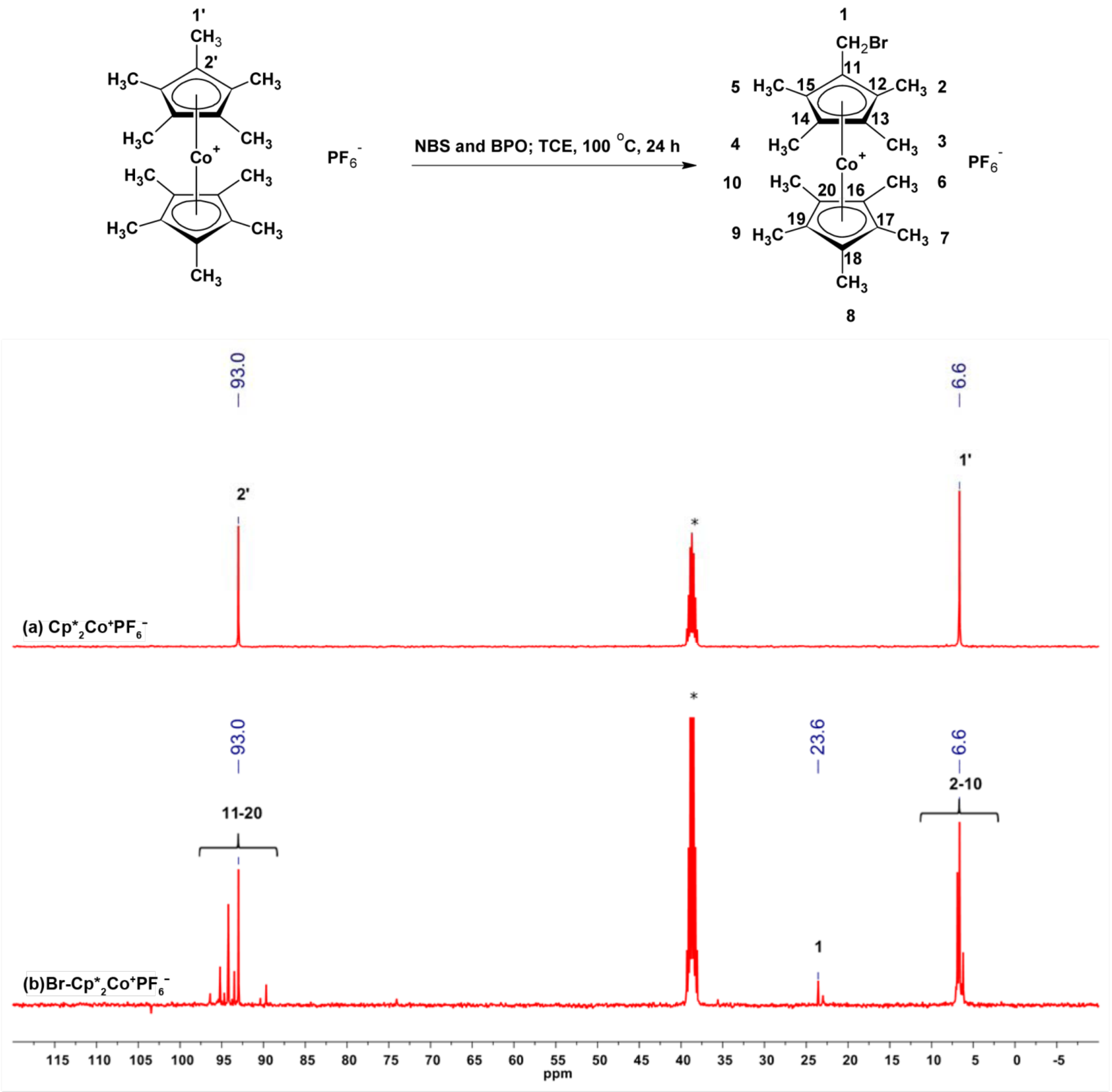

Fig. S12. ^{13}C NMR spectroscopy evidence for bromination of permethyl cobaltocenium hexafluorophosphate (Br-$Cp^*_2Co^+PF_6^-$). (**a**) Before bromination ($Cp^*_2Co^+PF_6^-$). (**b**) After bromination (Br-$Cp^*_2Co^+PF_6^-$). DMSO-d_6 was used as solvent (39.52 ppm, labeled "*"). $Cp^*_2Co^+PF_6^-$ shows characteristic peaks for ring (93.0 ppm) and methyl carbons (6.6 ppm). After bromination, a new peak for the bromomethyl carbon appears (23.6 ppm), perturbing both nearby ring and methyl carbons but leaving them at essentially the same chemical shifts. Similar perturbation is also observed in ^{1}H (**Fig. S8**) and ^{19}F (**Fig. S9**) NMR spectroscopy. These results are consistent with ^{1}H NMR spectroscopy (**Fig. S8**) and mass spectroscopy (**Fig. S13**) as further confirmation.

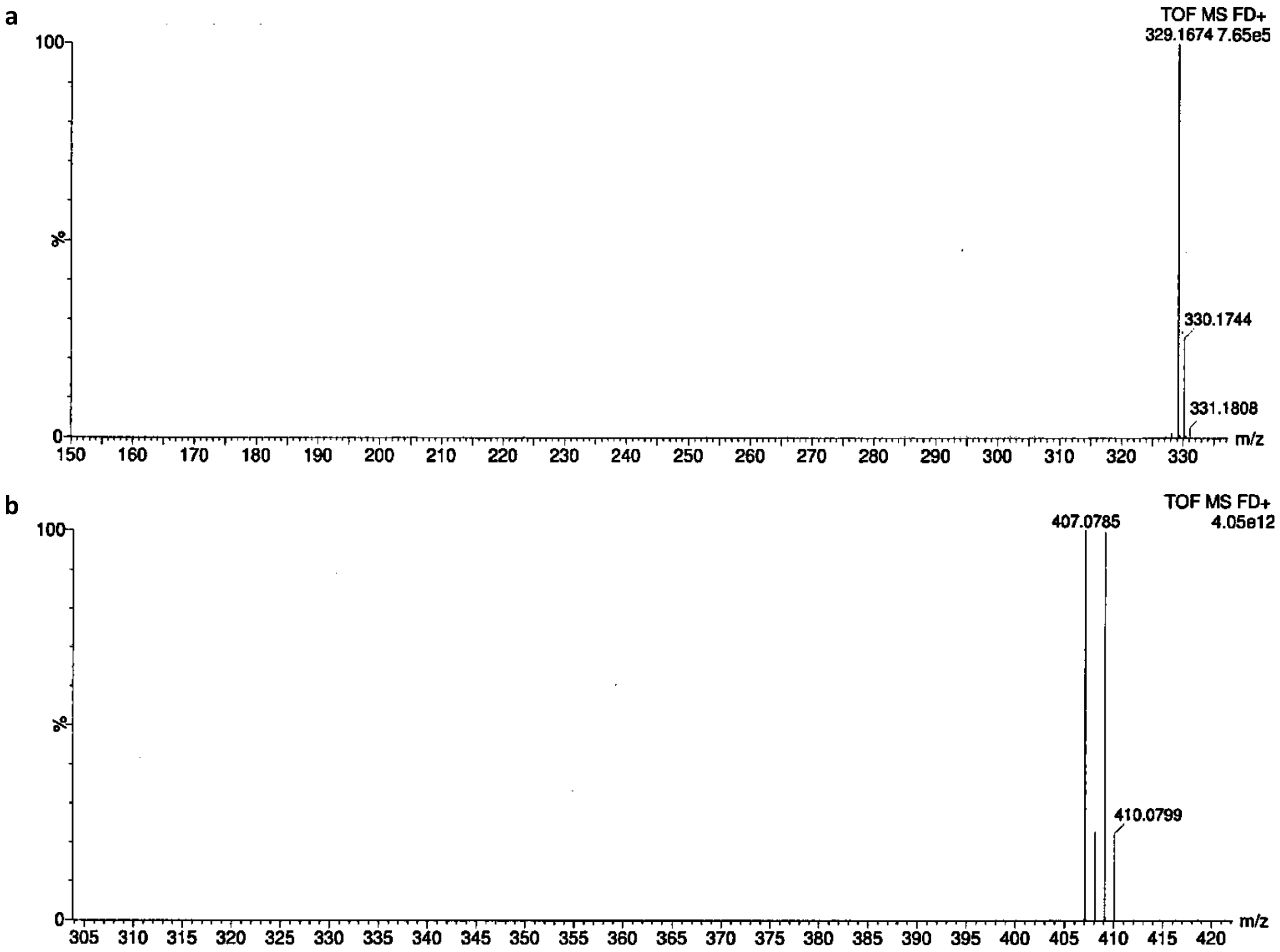

Fig. S13. Mass spectroscopy (MS) evidence for bromination of permethyl cobaltocenium ($Cp^*{}_2Co^+$). (**a**) Before bromination ($Cp^*{}_2Co^+PF_6^-$, expected m/z: 329.39 for $Cp^*{}_2Co^+$). (**b**) After bromination and anion exchange (Br-$Cp^*{}_2Co^+Cl^-$, expected m/z: 408.29 for Br-$Cp^*{}_2Co^+$). A Waters GCT Premier high-resolution time-of-flight mass spectrometer with liquid injection field desorption ionization (LIFDI) was used. The analyte was applied to the filament in dichloromethane, which was allowed to evaporate before ramping to a 12K voltage field. The sample was ionized at 30 mA. $Cp^*{}_2Co^+PF_6^-$ showed a peak at m/z = 329.17, matching the expected value of 329.39; after bromination, Br-$Cp^*{}_2Co^+PF_6^-$ showed a peak at m/z = 407−410, matching the expected value of 408.29. The small variation peak position is due to the existence of heavier isotopes, particularly for Br after bromination. These results are consistent with ^1H (**Fig. S8**) and ^{13}C (**Fig. S12**) NMR spectroscopy results. No sign of multiple bromination was detected.

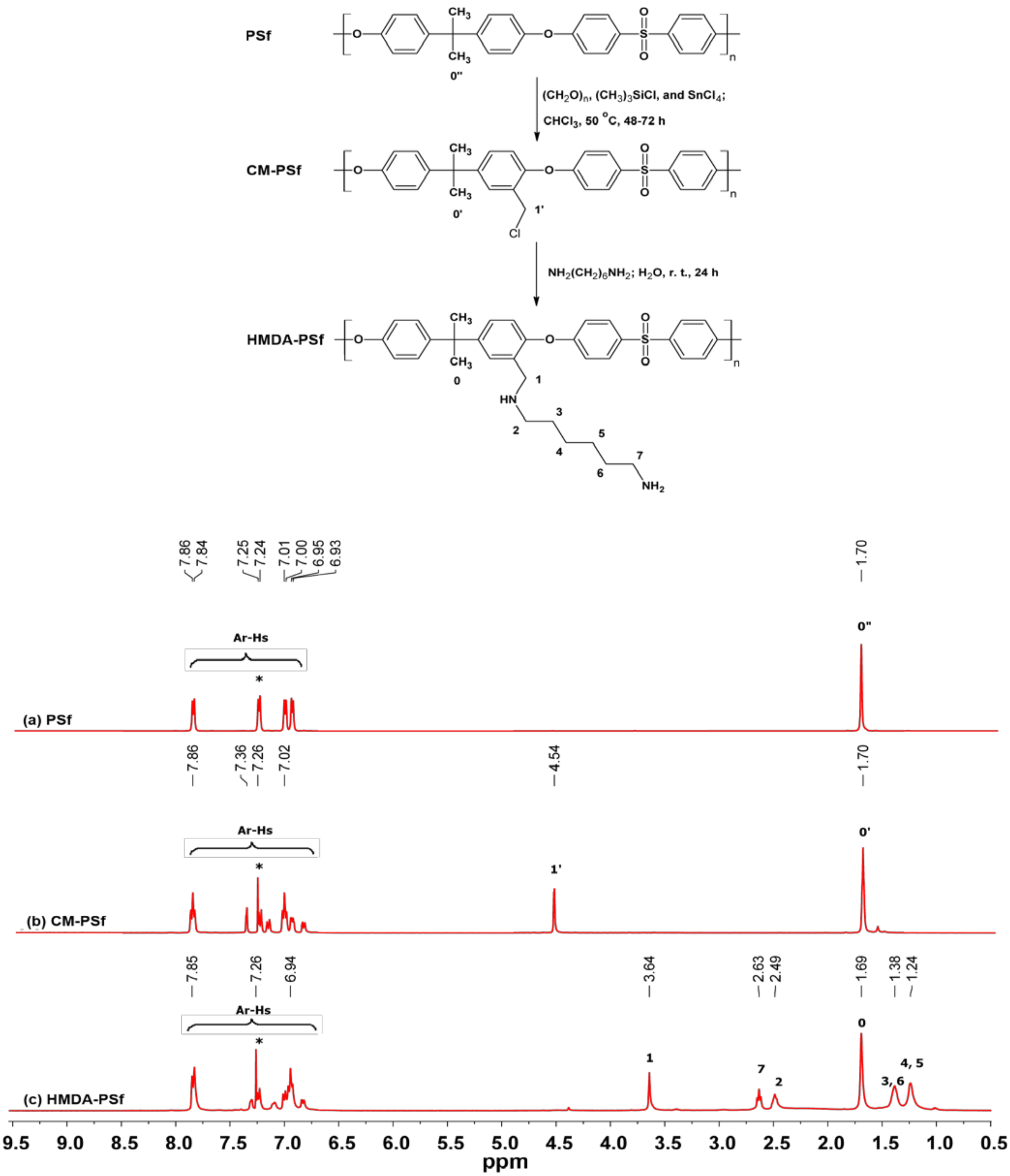

Fig. S14. ^{1}H NMR spectroscopy evidence for synthesis of hexamethylenediamine-aminated polysulfone (HMDA-PSf). (**a**) Polysulfone (PSf). (**b**) Chloromethylated polysulfone (CM-PSf). (**c**) Hexamethylenediamine-aminated polysulfone (HMDA-PSf). $CDCl_3$ was used as solvent (7.26 ppm, labeled "*").

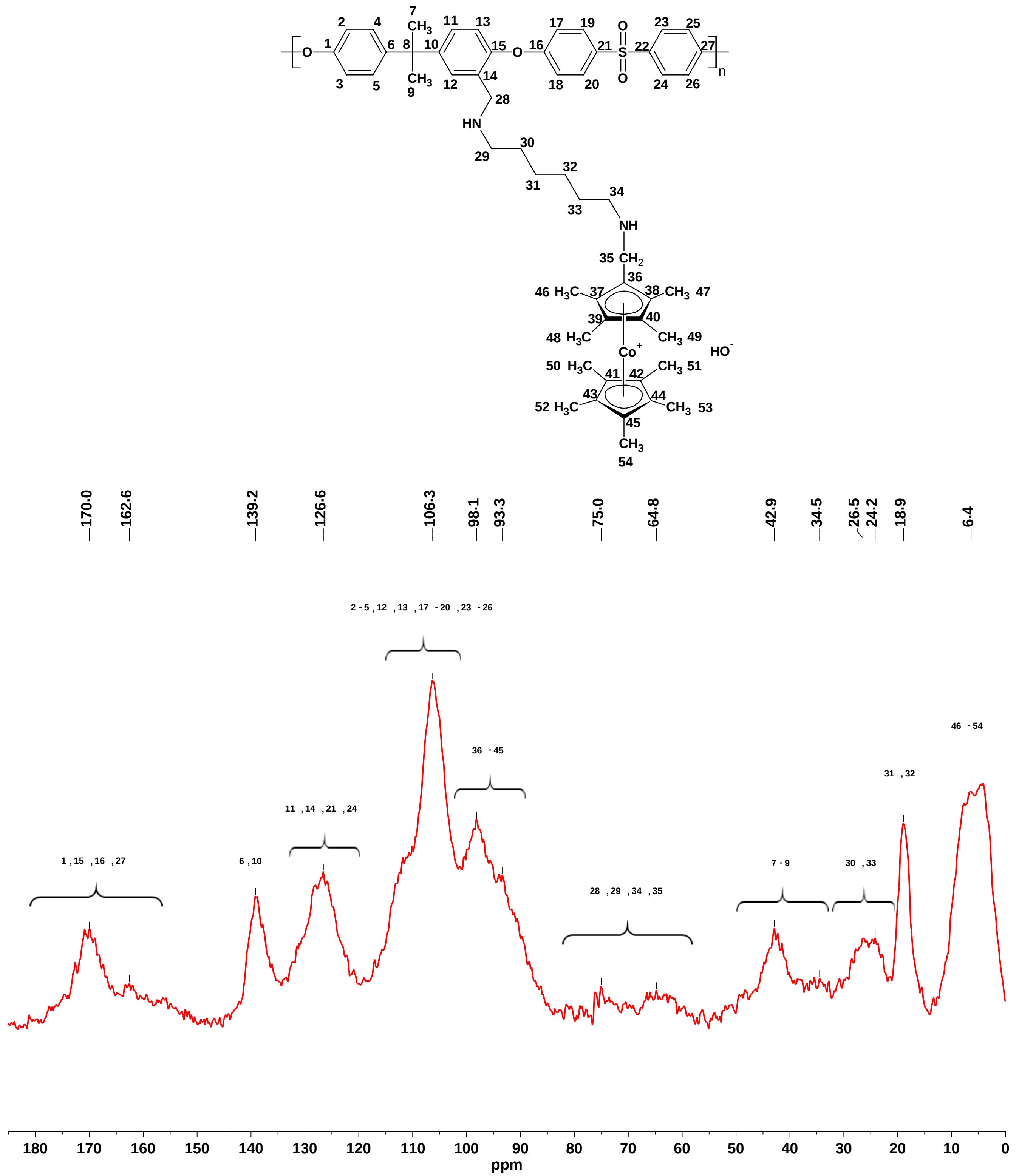

Fig. S15. Solid-state ^{13}C NMR spectroscopy of permethyl cobaltocenium-functionalized polysulfone hydroxide ($Cp^*_2Co^+$-PSf). The chemical shifts of ring (93−98 ppm) and methyl carbons (around 6.4 ppm) are consistent (93.4 and 6.3 ppm, respectively) with those of $Cp^*_2Co^+$ small-molecule salts in the literature(3) and this work (**Fig. S12**).

a

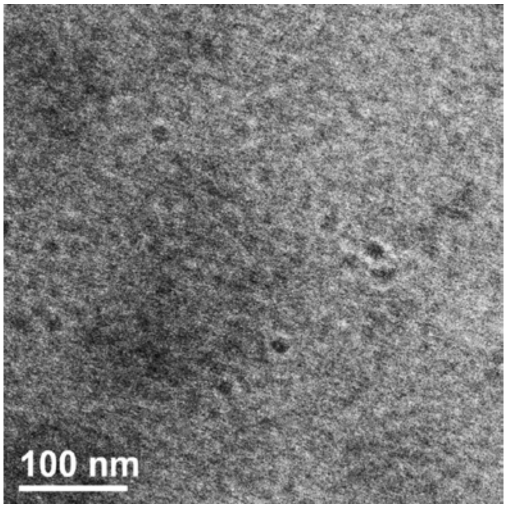

b

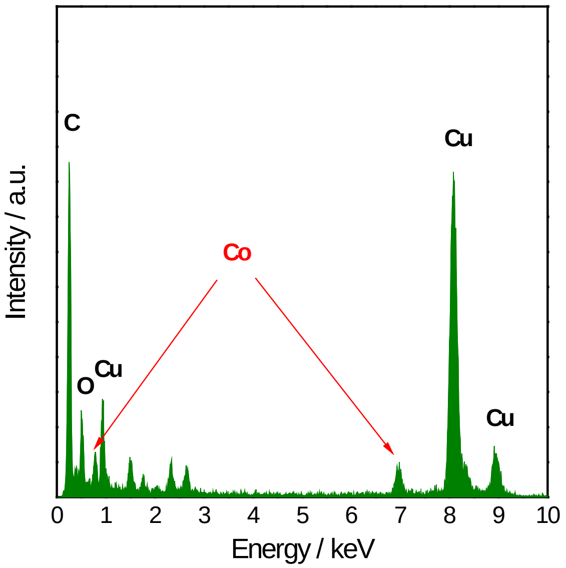

Fig. S16. Micromorphology of $Cp^{*}_{2}Co^{+}$-PSf membrane. (**a**) TEM image. (**b**) EDX spectrum taken during imaging. In the EDX spectrum, cobalt element was identified as expected and copper was present from copper grid.

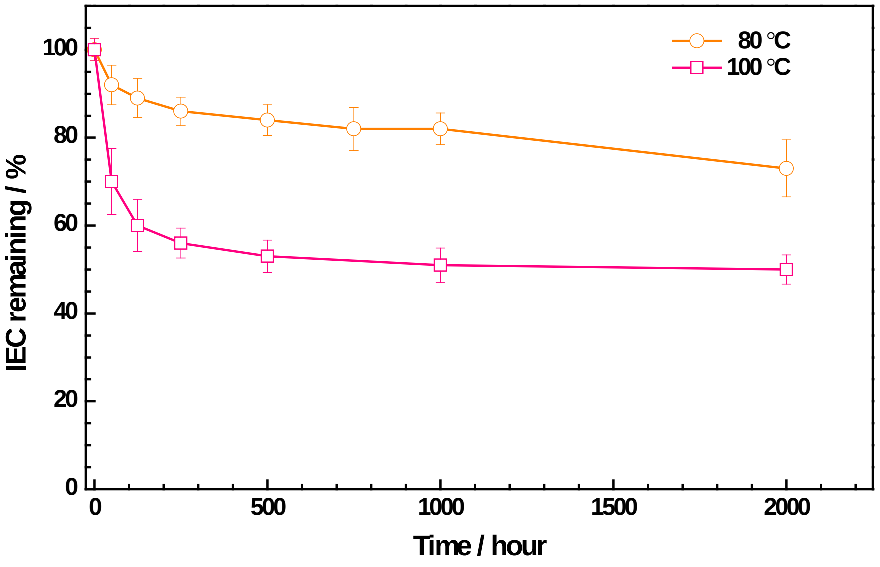

Fig. S17. The IEC change of $Cp^{*}{}_{2}Co^{+}$-PSf membranes versus time during the alkaline stability test. Test conditions: 1 M KOH as test alkaline solution; and the ratio of the membrane weight to the amount of test solution as 1 g vs. 200 mL. Orange curve: 80 °C test and purple curve: 100 °C test.

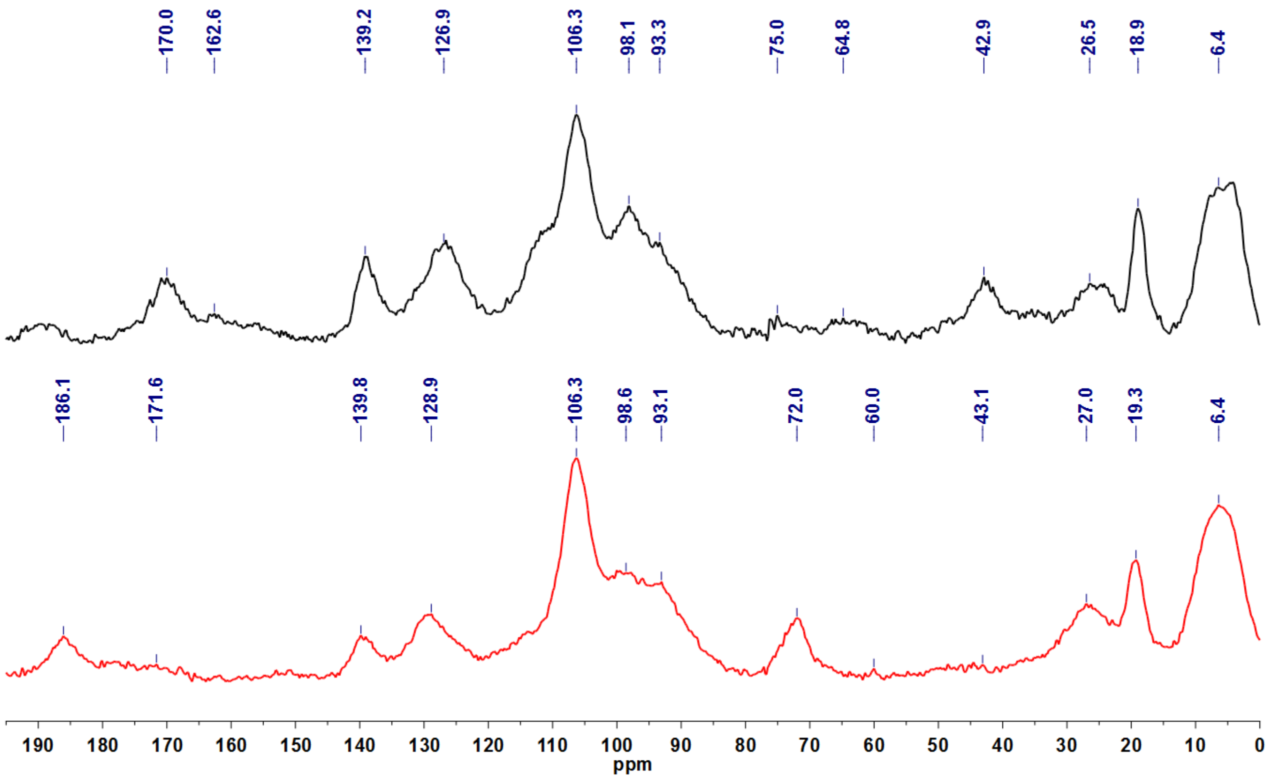

Fig. S18. Solid-state ^{13}C NMR spectra of $Cp^{*}_{2}Co^{+}$-PSf membrane before and after the alkaline stability test (80 °C, 1 M KOH, 6 weeks or 1,000 hours). Before test: black curve (top); after test: red curve (bottom). Test condition: 80 °C, 1 M KOH, 6 weeks or 1,000 hours. The chemical shifts of ring (93−98 ppm) and methyl carbons (~6.4 ppm) of permethyl cobaltocenium groups had almost no change after the stability test, consistent with the excellent stability of the small-molecules (**Fig. S2**). Most of chemical shifts (peaks 106.3−139.2 ppm) of PSf backbone remained at the same positions and similar intensities without significant changes. Chemical shifts (peaks ~19 and ~27 ppm) of the middle four carbons (numbers 30, 31, 32, and 33, seen in **Fig. S15**) of hexamethylene bridge had also no substantial change during the stability test, suggesting unchanged linkers between PSf backbone and permethyl cobaltocenium groups. Note: although Cp_2Co^+ functional groups remained almost unchanged, there are some signs of PSf backbone's degradation. One peak at 42.9 ppm (−**C**(CH_3)$_2$− in PSf) before stability test moved to 72.0 ppm after stability test, suggesting the formation of −**C**(CH_3)$_2$OH group; Another peak at 170.0 ppm (alpha-C of aromatic ether in PSf) before stability test moved to 186.1 ppm after stability test, suggesting the formation of quinone group (alpha-C). The shifts of the two peaks can result from PSf backbone scission, which provides insights into the loss of membrane performance. Basically, the post-stability solid-state ^{13}C NMR spectroscopy analysis is in agreement with the post-stability IEC results.

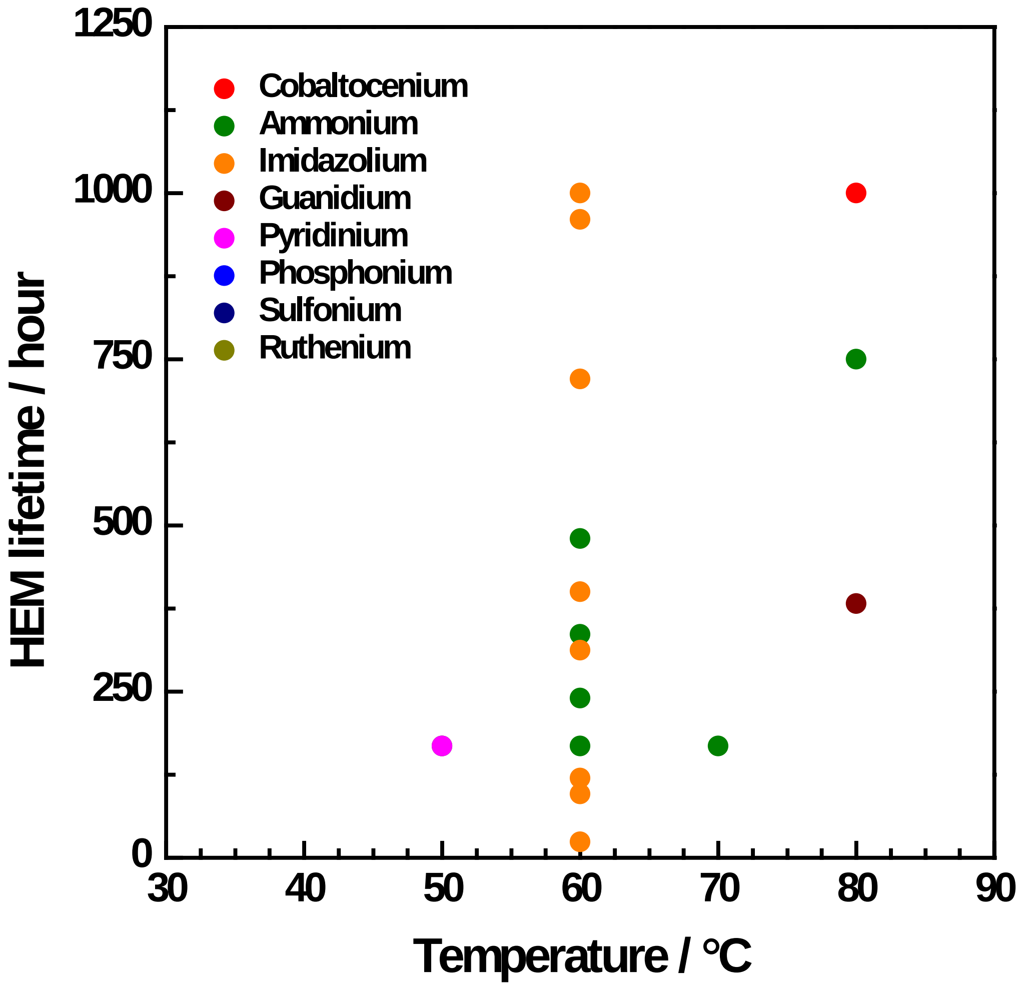

Fig. S19. Alkaline stability of $Cp^*_2Co^+$-PSf and other HEMs. Test conditions: 1 M KOH or NaOH as test medium and 20% IEC loss as degradation threshold, or otherwise noted. Cobaltocenium: $Cp^*_2Co^+$-functionalized PSf (this work). Ammonium: *btm*Am-based PETFE,(8) PSt,(9) poly(styrene acrylonitrile),(10) and PETFE;(8) *badm*Am-based PPO.(11) Imidazolium: *adm*Im-based PFS;(12) *bm*Im-based Poly(styrene acrylonitrile);(10) *aam*Im-based crosslinked PStAE;(13) *am*Im-based crosslinked PStAE(10); *bdm*Im-based PETFE;(8) *dmtmp*BIm-based PBI (2 M KOH).(14) Guanidinium: *ppm*Gu-based fPSf (0.5 M NaOH);(15) Pyridinium: *a*Py-based PSt (0.1 M KOH).(9) Full names of the cations are shown in **Table S3**. Note that a test duration of at least 24 hours was required for inclusion. Caution is needed in comparing the stability of hydroxide-exchange membranes with different cations and backbones, because both cation and backbone have impacts on alkaline stability of the membrane.

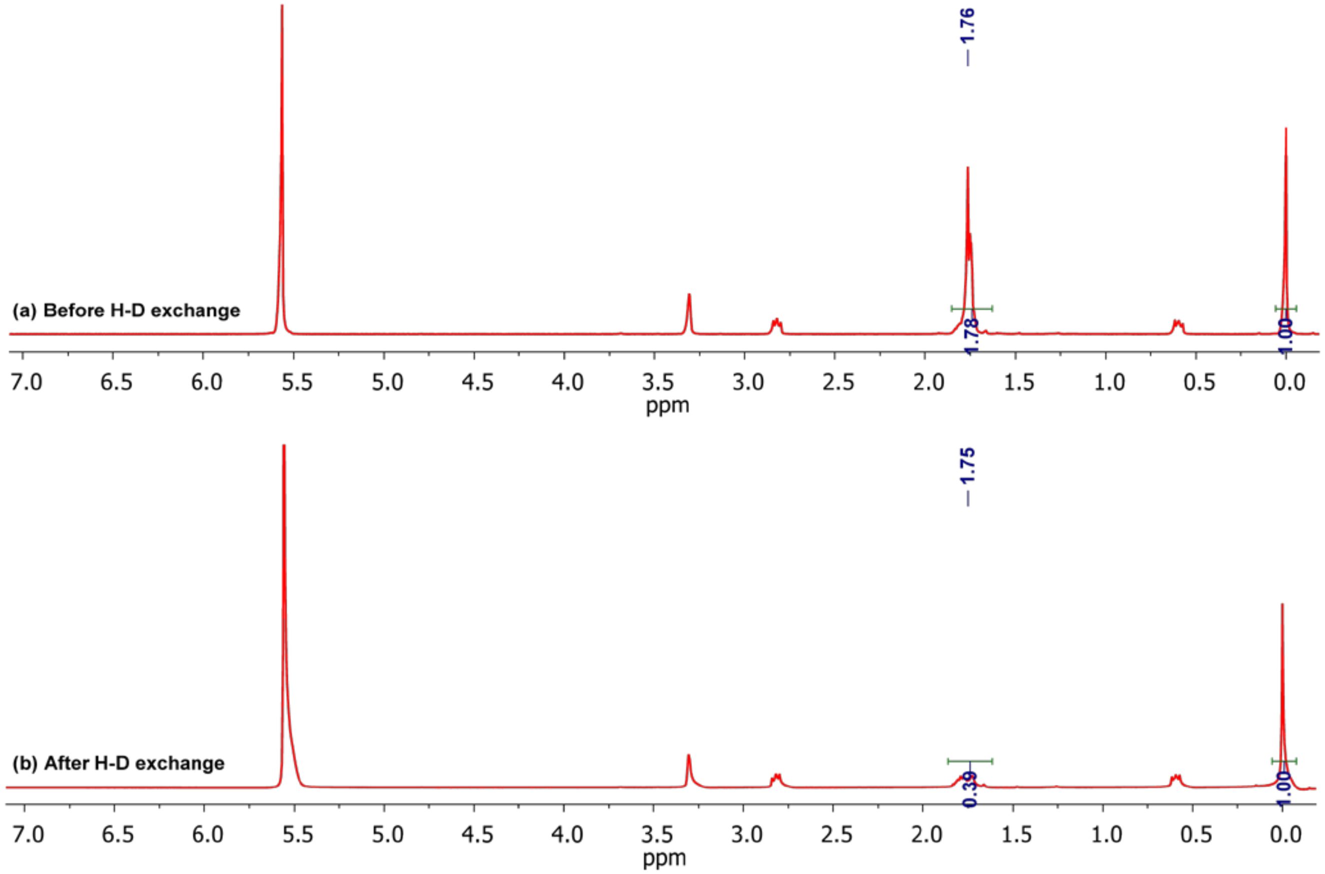

Fig. S20. ^{1}H NMR spectroscopy evidence for H-D isotopic exchange of methyl protons in permethyl cobaltocenium ($Cp^*_2Co^+OH^-$). (**a**) Before H-D exchange. (**b**) After H-D exchange. Test conditions: 60 °C, 30 min, 40% KOH/D_2O in methanol (1.42 g in 10 ml), 3-(trimethylsilyl)-propane sulfonic sodium as internal standard (2.90, 1.75, 0.60, and 0 ppm). The degree of H-D exchange (DE) can be calculated by the equation: $DE = [1-(A1/A0)_f/(A1/A0)_i] \times 100\%$, where A1 and A0 are the integrals of the methyl peak (1.76 ppm) and the internal standard peak (0 ppm), respectively. Subscripts "i" and "f" represent before and after the test, respectively. The results show a degree of H-D exchange of 78%.

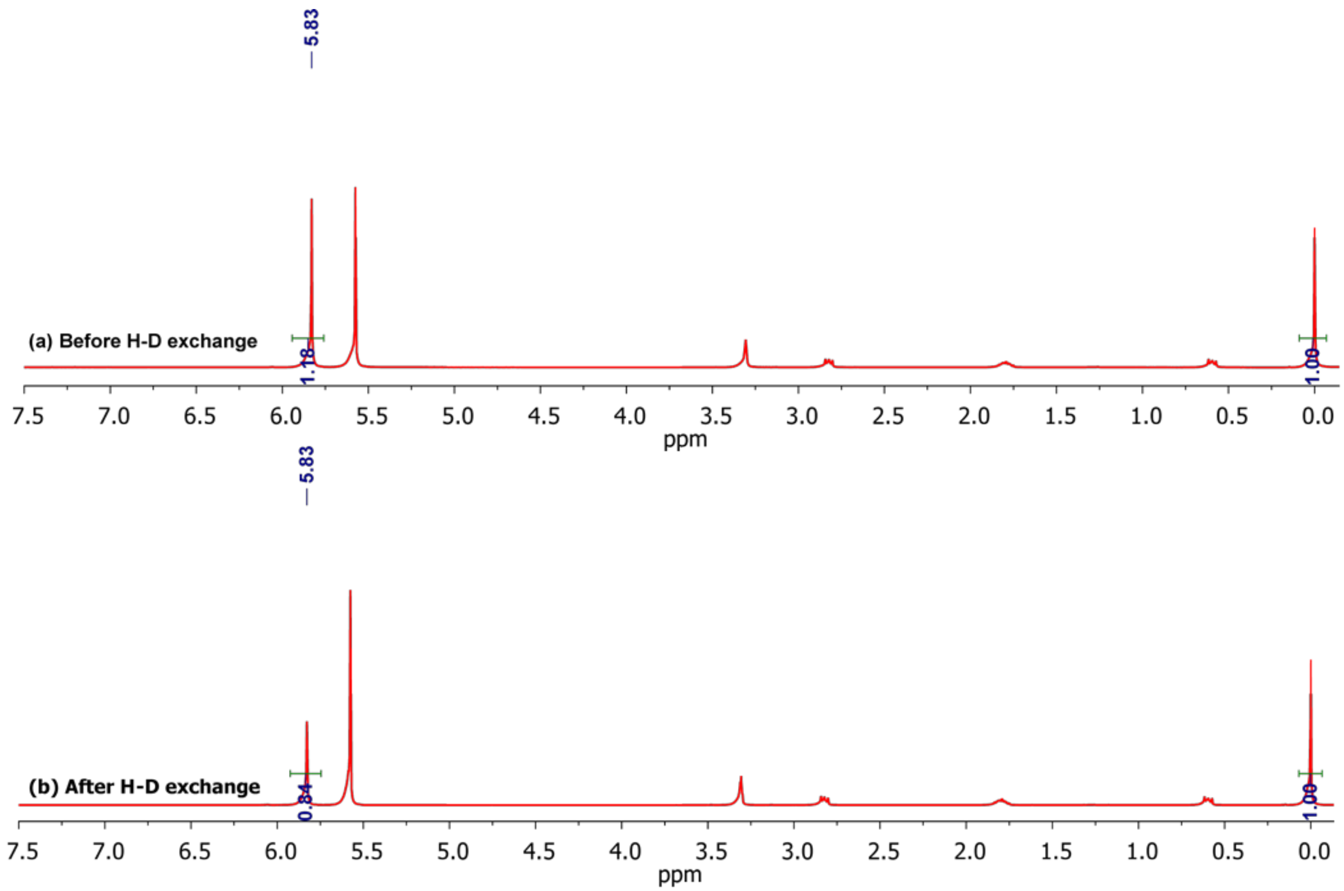

Fig. S21. ^{1}H NMR spectroscopy evidence for H-D isotopic exchange of ring protons in parent cobaltocenium ($Cp_2Co^+OH^-$). (**a**) Before H-D exchange. (**b**) After H-D exchange. Test conditions: see **Fig. S20**. The degree of H-D exchange (DE) can be calculated by the equation: DE = $[1-(A1/A0)_f/(A1/A0)_i] \times 100\%$, where A1 and A0 are the integrals of the ring peak (5.83 ppm) and the internal standard peak (0 ppm), respectively. Subscripts “i” and “f” represent before and after the test, respectively. The results show a degree of H-D exchange of 29%.

References

1. Gu S, *et al.* (2009) A Soluble and Highly Conductive Ionomer for High-Performance Hydroxide Exchange Membrane Fuel Cells. *Angew Chem Int Edit* 48(35):6499-6502.
2. Gu S, *et al.* (2006) Synthesis and characteristics of sulfonated poly(phthalazinone ether sulfone ketone) (SPPESK) for direct methanol fuel cell (DMFC). *J Membrane Sci* 281(1-2):121-129.
3. Robbins JL, Edelstein N, Spencer B, & Smart JC (1982) Syntheses and Electronic-Structures of Decamethylmetallocenes. *J Am Chem Soc* 104(7):1882-1893.
4. Occelli ML & Kessler H (1996) *Synthesis of Porous Materials: Zeolites: Clays, and Nanostructures* (Marcel Dekker, Inc, New York, New York).
5. Kondo M, *et al.* (2004) A new redox-active coordination polymer with cobalticinium dicarboxylate. *Inorg Chem* 43(19):5801-5803.
6. Haddad B, Villemin D, Belarbi E, Bar N, & Rahmouni M (in press) New dicationic piperidinium hexafluorophosphate ILs, synthesis, characterization and dielectric measurements. *Arabian Journal of Chemistry*.
7. Vygodskii YS, *et al.* (2008) Conductive polymer electrolytes derived from poly(norbornene)s with pendant ionic imidazolium moieties. *Macromol Chem Physic* 209(1):40-51.
8. Page OMM, *et al.* (2013) The alkali stability of radiation-grafted anion-exchange membranes containing pendent 1-benzyl-2,3-dimethylimidazolium head-groups. *RSC Adv* 3(2):579-587.
9. Hnat J, Paidar M, Schauer J, Zitka J, & Bouzek K (2011) Polymer anion selective membranes for electrolytic splitting of water. Part I: stability of ion-exchange groups and impact of the polymer binder. *J Appl Electrochem* 41(9):1043-1052.
10. Qiu B, Lin BC, Qiu LH, & Yan F (2012) Alkaline imidazolium- and quaternary ammonium-functionalized anion exchange membranes for alkaline fuel cell applications. *J Mater Chem* 22(3):1040-1045.
11. Li NW, Leng YJ, Hickner MA, & Wang CY (2013) Highly Stable, Anion Conductive, Comb-shaped Copolymers for Alkaline Fuel Cells. *J Am Chem Soc* 135(27):10124-10133.
12. Lin BC, Qiu LH, Qiu B, Peng Y, & Yan F (2011) A Soluble and Conductive Polyfluorene Ionomer with Pendant Imidazolium Groups for Alkaline Fuel Cell Applications. *Macromolecules* 44(24):9642-9649.
13. Qiu B, *et al.* (2012) Bis-imidazolium-based anion-exchange membranes for alkaline fuel cells. *J Power Sources* 217:329-335.
14. Thomas OD, Soo KJWY, Peckham TJ, Kulkarni MP, & Holdcroft S (2012) A Stable Hydroxide-Conducting Polymer. *J Am Chem Soc* 134(26):10753-10756.
15. Kim DS, Labouriau A, Guiver MD, & Kim YS (2011) Guanidinium-Functionalized Anion Exchange Polymer Electrolytes via Activated Fluorophenyl-Amine Reaction. *Chem Mater* 23(17):3795-3797.
